# Translation of mouse model to human gives insights into periodontitis etiology

**DOI:** 10.1038/s41598-020-61819-0

**Published:** 2020-03-17

**Authors:** Aysar Nashef, Munz Matthias, Ervin Weiss, Bruno G. Loos, Søren Jepsen, Nathalie van der Velde, André G. Uitterlinden, Jürgen Wellmann, Klaus Berger, Per Hoffmann, Matthias Laudes, Wolfgang Lieb, Andre Franke, Henrik Dommisch, Arne Schäfer, Yael Houri-Haddad, Fuad A. Iraqi

**Affiliations:** 1 Department of Prosthodontics, Dental school, The Hebrew University, Hadassah Jerusalem, Israel; 2 Department of Oral and Maxillofacial surgery, Poriya Medical center, Poriya, Israel; 3 Department of Periodontology and Synoptic Medicine, Institute for Dental and Craniofacial Sciences, Charité – University Medicine Berlin, Berlin, Germany; 4 Institute for Cardiogenetics, University of Lübeck, 23562 Lübeck, Germany; 5 School of Dental Medicine, Tel-Aviv University, Tel-Aviv, Israel; 6 Department of Periodontology and Oral Biochemistry, Academic Centre for Dentistry Amsterdam (ACTA), University of Amsterdam and Vrije Universiteit Amsterdam, Amsterdam, The Netherlands; 7 Department of Periodontology, Operative and Preventive Dentistry, University of Bonn, Bonn, Germany; 8 Department of Internal Medicine, Erasmus Medical Center, Rotterdam, the Netherlands; 9 Department of Internal Medicine section of Geriatrics, Amsterdam Medical Center, Amsterdam, The Netherlands; 10 Institute of Epidemiology and Social Medicine, University Münster, Münster, Germany; 11 Institute of Human Genetics, University of Bonn, Bonn, Germany; 12 Human Genomics Research Group, Department of Biomedicine, University Hospital of Basel, Basel, Switzerland; 13 Department of Medicine, University of Kiel, Kiel, Germany; 14 Institute of Epidemiology, Christian-Albrechts-University, Kiel, Germany; 15 Institute of Clinical Molecular Biology, Christian-Albrechts-University, Berlin, Germany; 16 Department of Clinical. Microbiology and Immunology, Faculty of Medicine, Tel-Aviv University, Tel-Aviv, Israel

**Keywords:** Dental diseases, Health care

## Abstract

To suggest candidate genes involved in periodontitis, we combined gene expression data of periodontal biopsies from Collaborative Cross (CC) mouse lines, with previous reported quantitative trait loci (QTL) in mouse and with human genome-wide association studies (GWAS) associated with periodontitis. Periodontal samples from two susceptible, two resistant and two lines that showed bone formation after periodontal infection were collected during infection and naïve status. Differential expressed genes (DEGs) were analyzed in a case-control and case-only design. After infection, eleven protein-coding genes were significantly stronger expressed in resistant CC lines compared to susceptible ones. Of these, the most upregulated genes were *MMP20* (P = 0.001), *RSPO4* (P = 0.032), *CALB1* (P = 1.06×10^−4^), and *AMTN* (P = 0.05). In addition, human orthologous of candidate genes were tested for their association in a case-controls samples of aggressive (AgP) and chronic (CP) periodontitis (5,095 cases, 9,908 controls). In this analysis, variants at two loci, *TTLL11/PTGS1* (rs9695213, P = 5.77×10^−5^) and *RNASE2* (rs2771342, P = 2.84×10^−5^) suggested association with both AgP and CP. In the association analysis with AgP only, the most significant associations were located at the HLA loci HLA-*DQH1* (rs9271850, P = 2.52×10^−14^) and HLA-*DPA1* (rs17214512, P = 5.14×10^−5^). This study demonstrates the utility of the CC RIL populations as a suitable model to investigate the mechanism of periodontal disease.

## Introduction

Periodontitis (PD) is one of the most common complex inflammatory diseases in human. The disease is believed to be a multifactorial trait which initiated by compositional shift of the oral micro biome because of different stimuli resulting in destruction of tissues surrounding the teeth. The precise mechanisms underlying individual disease susceptibility or that drive the individual steps in the pathogenesis of PD have largely remained unknown. Previous genome-wide association studies (GWAS) of chronic periodontitis (CP) reported several ‘suggestive’ susceptibility loci but failed to produce genome-wide significant evidence of association^1–6^. GWAS that used the rare but severe and early-onset form aggressive periodontitis (AgP), which is believed to have a stronger genetic component, or studies that combined AgP and CP analysis samples to increase the statistical power, identified five genome-wide significant loci^7–9^. Compared to other complex diseases, the low number of statistically significant genetic risk loci likely reflects the relatively small sample sizes of the available PD cohorts. Given these size limitations and the general scarcity of high-quality phenotype-genotype cohorts for PD, GWAS alone are unlikely to identify the missing susceptibility loci. Here, the mouse model provides an appropriate and powerful system to identify genes that have a role in the etiology of complex human diseases. Given that expression patterns in mouse models recapitulate those in humans^10^, the examination of differences in gene expression as a response to different environmental exposures in genetically identical mice and between genetically different mice living in the same environmental context, can give direct insight into the mechanism that lead to disease susceptibility. Additionally, the genes underlying these differences can be mapped as quantitative trait loci (QTL)^11^. These QTLs and the differently expressed genes (DEGs) can be translated to the human orthologous genes and serve as candidates for genetic association studies in human case-control samples. Recently, we demonstrated the strength of combining genome-wide expression profiling and QTL-mapping in a F_2_ mouse population and identified the human chromosomal region at *PF4/PPBP/CXCL5* to carry genetic susceptibility variants of AgP and CP^12^. In the current study, we used recombinant inbred mouse lines of the Collaborative Cross (CC) that were descended from eight divergent strains of mice: A/J, C57BL/6 J, 129S1/SvImJ, NOD/LtJ, NZO/HiLtJ, CAST/Ei, PWK/PhJ, and WSB/EiJ and specifically designed for high-resolution mapping of QTLs^13^. Briefly, this population provide an increased level of genetic and phenotypic diversity compared to other existing mouse genetic reference populations^14^. The full description of the CC population under development in our labs at Tel-Aviv University and its power for dissecting complex traitsis described in recent publications^13,15–20^. In previous studies, we identified CC-RILs with increased susceptibility and resistance to PD as a result of mixed infection with the oral bacteria *Porphyromonas gingivalis (P.g)* and *Fusobacterium nucleatum (F.n)*^21^ and mapped two QTLs that were associated with alveolar bone loss in the CC mouse population^22^. In the current study, we hypothesized that in response to an identical challenge of bacterial infection, resistant and susceptible CC-RILs have different gene expression profiles. The identification of the significant DEGs could pinpoint putative molecular pathways and mechanisms that contribute to disease resistance and susceptibility. These genes also provide reasonable candidate genes for association studies in human case-control analyses populations of PD. To test our hypothesis, we investigated the expression patterns of oral tissues in six susceptible and resistant CC-RILs before and after mixed infection with *P.g*. and *F.n*.^21^. We identified a set of genes to be significantly upregulated in resistant CC-RILs after bacterial infection. We present new data that support the previously suggested susceptibility genes *TTLL11*, HLA-*DOA* and *LBP*, and propose HLA-*DQH1* and *RNASE2* as novel risk factors for AgP and PD.

## Results

### Gene enrichment analysis

**Figure 1:**
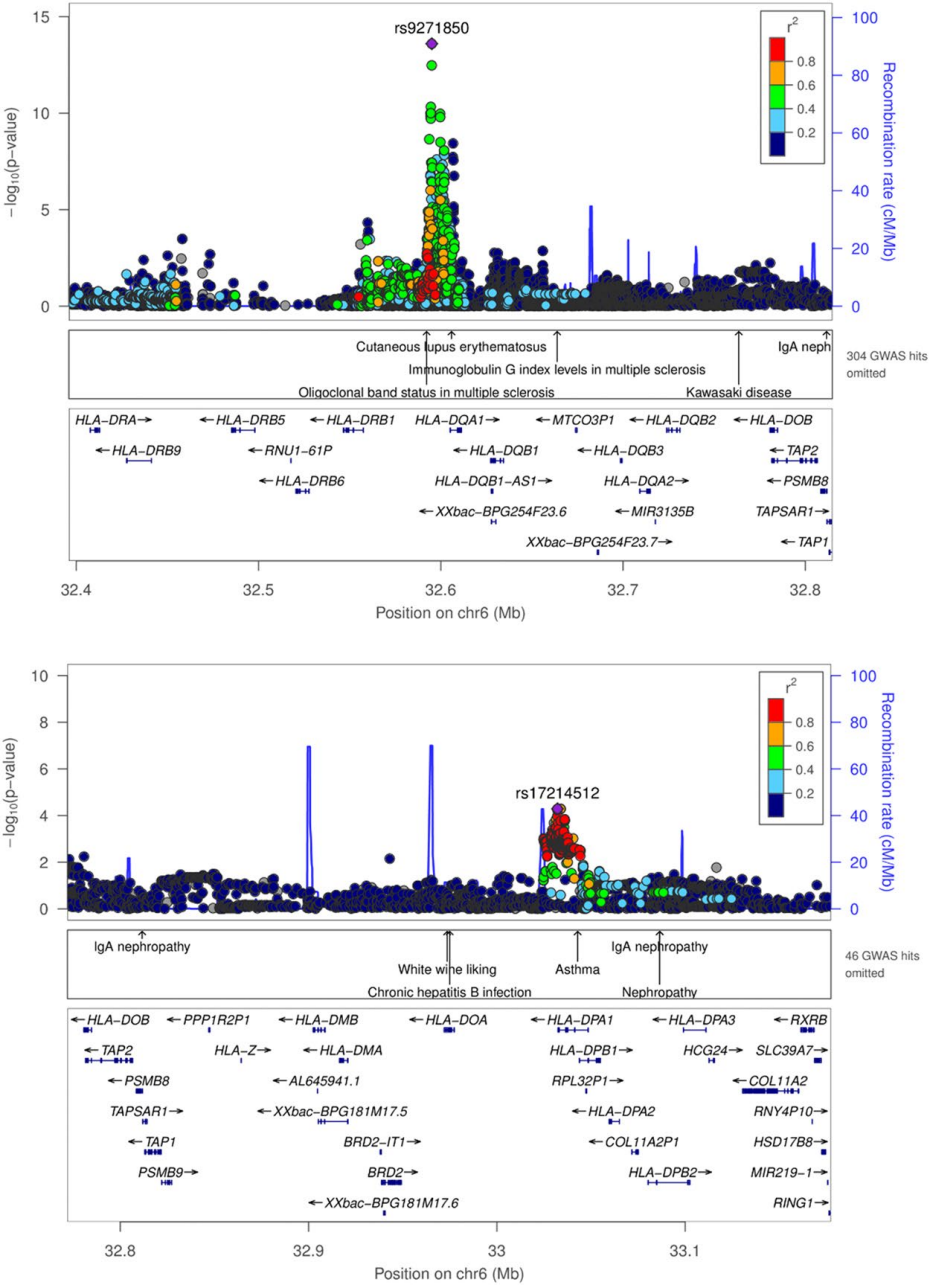
Regional association plots of the most significant associations with AgP case-control cohort that located at the HLA loci, (**A**) HLA-*DQH1* and (**B**) HLA-*DPA1*.

The Principle Component Analysis (PCA) showed that at 42 days after infection, the largest variation within the 24 samples was between the genetically different RILs (e.g. resistant vs. susceptible vs. bone formation group) rather than between the infection/control conditions **(**Appendix-Fig. 1**)**. All of RNAseq raw data were submitted to GEO website for allowing freely public access. The accession number of the raw data is **GSE145474**, and can be access by website: https://www.ncbi.nlm.nih.gov/geo/query/acc.cgi?acc=GSE145474.

### Differential gene expression in periodontal tissues in susceptible, resistant, and bone-formation RILs after oral bacterial infection compared to mock-infection

For each of the three phenotypes, we tested the differential gene expression between infected vs. non-infected individuals (Appendix-Table 1). This analysis showed how each of the three groups specifically responded to bacterial infection on the transcription level, giving a potential mechanistic link between the phenotype and specific DEGs. Eleven DEGs were observed in the phenotype “resistant”, and two within the phenotype “susceptible”. No significant DEG was observed for the phenotype “bone formation”. All DEGs except of *Bpifa6* could be mapped to an orthologous autosomal gene in the human genome (*Xist* is a non-coding RNA gene that is located on the X-chromosome. This chromosome was excluded from the human GWAS data set and the subsequent analyses).*Obp2b* showed homology with the two human genes *OBP2A* and *OBP2B* **(**Appendix-Table 1).

### Differential gene expression in periodontal tissues between the susceptible, resistant and bone-formation RILs after oral bacterial infection

**Figure 2:**
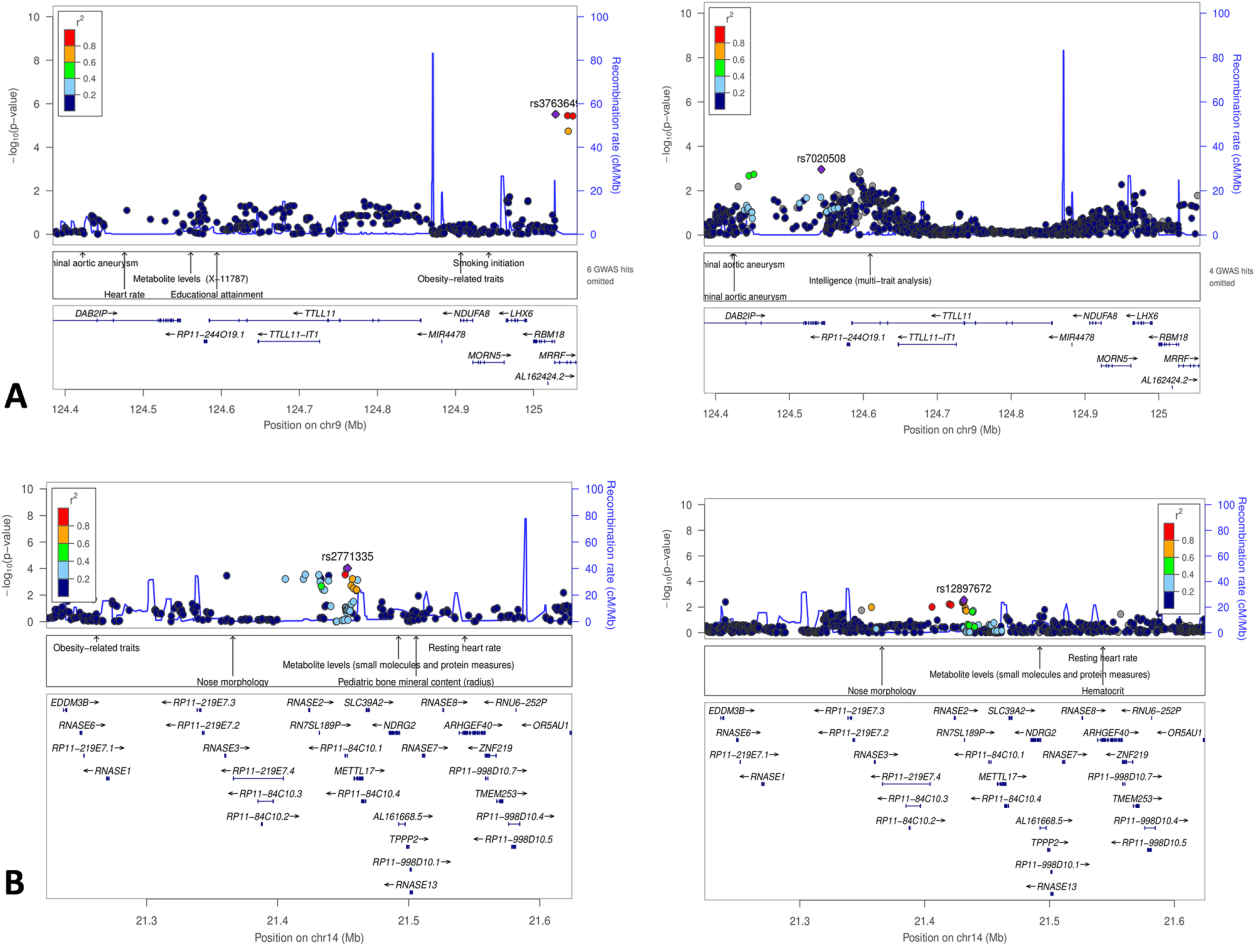
Regional association plots of the loci *DAB2IP/TTLL11* and *RNASE2*, which suggested association in the meta-analyis of AgP and CP with p < 10^−04^. (**A**) *DAB2IP/TTLL11*. Left panel**:** The association upstream *TTLL11*, tagged by rs3763649, was observed in the discovery meta-analysis using AgP and CP cases and controls. Right panel: After exclusion of the CP case-control samples, the associated region was identical to the genetic locus associated with increased quantities of *P.G*. in humans (Red rectangle = region associated with increased quantities of *P.G*. in humans. (**B**) *RNASE2*. Left panel**:** The association of the suggested novel susceptibility locus of PD was strongest in the meta-analysis using AgP and CP cases and controls. Right panel: After exclusion of the CP case-control samples, the associated region was less significant, possibly reflecting the reduced statistical power.

We next analyzed the differences in gene expression between the three genetically different phenotype groups after bacterial infection. The gene expression after bacterial infection of the CC-RILs of one phenotype group was compared with the gene expression after bacterial infection of the CC-RILs of the other two-phenotype groups. 1,326 genes were differentially expressed between infected susceptible and resistant RILs, 823 genes were differentially expressed between infected susceptible RILs compared to infected RILs of the bone formation, and 1,572 genes were differentially expressed between infected resistant RILs compared to the bone formation group (Padj < 0.05, log2FC > 1). Heatmaps of the expressed values for each comparison are shown in Appendix-Fig. 2. The ten strongest up regulated and the ten strongest down-regulated genes of each comparison are shown in Appendix Table 2. The regulated genes in the susceptible group were significantly related to immune response pathways FDR < 0.05 as shown in Appendix-Fig. 3. Functional network analyses of DEGs within the observed pathways are shown in Appendix Figs. 4, -5 and -6.

To nominate candidate genes that have a role in the etiology of PD, we selected the ten most up- or downregulated DEGs, the DEGs that mapped to the QTLs *Perio*3 and *Perio4* or that were suggested as risk genes by published human GWAS on PD. The QTLs *Perio*3 and *Perio4* comprised 81 mouse genes **(**Appendix Table 3**)**. Six DEGs *(PARP1, DUSP23, PCDH17, PSEN2, H3f3aos, EPHX1*) located to the QTLs *Perio*3 and *Perio4* **(**Table 1, *H3f3oas* and *Dusp*23 have no human orthologous and showed only borderline significance with P = 0.07). 15 DEGs had been reported in previous human GWAS on periodontitis with P < 9 × 10^−6^ (*C1orf*87, *HLA-DOA, BIRC*5*, CCDC*13*, GLDC, GPR*141*, OTOF, IFI*16*, ETNK*2*, TTLL*11*, ATP*5*S, GRID*1*, LBP, NIN*, and *VAV*1; Appendix Table 4**)**. A potential gene regulatory network between QTLs associated with susceptibility to alveolar bone loss in CC mice with DEGs in susceptible vs. resistant lines and bone formation groups was proposed and shown in Appendix Figure 3; two genes within the significant QTL *Perio*3, *Psen*2 and *Parp1*, showed a HUB gene pattern.

**Table 1:** The table lists six genes and their Padj, which underlie the previous QTL (Perio3 and Perio4) and showed significant deferential expression between one or more of three comparisons.

Gene Name	Description	Chr.	Padj (Comparison)
Psen2	presenilin_2	1	0.037 (O/R)
Dusp23	dual_specificity_phosphatase_23	1	0.07 (O/R)
Ephx1	epoxide_hydrolase_1,_microsomal	1	0.01 (S/O)
H3f3aos	H3_histone,_family_3A,_opposite_strand	1	0.07 (S/R)
Pcdh17	protocadherin_17	14	0.09 (S/R); 0.04(O/R)
Parp1	poly_(ADP-ribose)_polymerase_family,_member_1	1	0.05 (O/R); 0.039 (S/O)

(O; bone formation group, S; susceptible group, R; resistant group).

### Candidate gene association study in case-control samples of AgP and CP

We selected the human orthologous genes of the 13 DEGs (and 200 kb up- and downstream to include putative regulatory sequences) that were identified after comparing the transcriptional response of the CC-RILs to bacterial infection with mock-infection. First, the common SNPs of these regions were examined for associations with the disease phenotype AgP. The smallest p-value was observed for a region upstream *OBP2A* (rs1329505, P = 7.85 x E-04), located ~73 kb upstream of SNP rs1537415 at *GLT6D1* that was previously reported to be associated with AgP with genome-wide significance^8^. Next, the variants at these loci were tested for association in a meta-analysis of AgP and CP. In this meta-analysis, the strongest association was observed for a large chromosomal region at *RPL29* (chr3:51,874,679–52,220,203; with p = 9.3 ×10E-05; rs16943). This region was previously reported to be associated with macrophage inflammatory protein 1b levels at a genome-wide significance level^23^. According to the GWAS Catalog, the tested genetic regions comprise 55 genome-wide significant associations and 61 suggestive associations with various phenotypes, as well as 5,903 eQTLs. These variants were tested for association in AgP and the meta-analysis of AgP and CP, if genotypes where available for both, the AgP and CP samples.

Next, we tested the associations of the common variants of the human orthologous genes (+ 200 kb up- and downstream) that corresponded to the ten most up- or downregulated DEGs, the DEGs that mapped to the QTLs *Perio*3 and *Perio4* or that were suggested as risk genes by published human GWAS on PD (Table 1**+** Appendix Table 2**+** Appendix Table 4). In total, 23,413 SNPs were tested for association with AgP (896 cases, 7,104 controls). In this analysis, 6,423 SNPs indicated an association with p < 0.05. The most significant associations were located at the HLA loci HLA-*DQH1* (rs9271850, P = 2,52^−14^) and HLA-*DPA1* (rs17214512, P = 5,14^−05^; Fig. 1, Table 2). In the AgP-CP meta-analysis that combined the AgP and CP samples (5.095 cases, 9.908 controls), the HLA loci lost significance. Instead, a genetic region at *TTLL11* (tubulin tyrosine ligase-like family, member 11), upstream *PTGS1* (prostaglandin G/H synthase 1), *(*chr9, rs9695213, *P* = 5.77 ×10^−5^ [in AgP only the association was P = 8.1 ×10^−3^]) and at *RNASE2* (Ribonuclease A Family Member 2) (chr14, rs2771342, *P* = 2.84 ×10^−5^ [in AgP only the association was not significant]) showed the most significant associations **(**Fig. 2, Table 2**)**. The chromosomal region at *TTLL11* was previously suggested to be associated with increased quantities of *P.g*. colonization of the human oral cavity before^1^. In addition to these loci, among the DEGs that mapped to the QTLs *Perio*3 and *Perio*4 and had additionally been suggested in previous human GWAS on PD as putative risk loci of PD, variants at *LBP* (chr.20) suggested association in the AgP-CP Meta-analysis (rs1780616, P = 1.67 ×10^−3^ [AgP; rs1780616, P = 7,78 ×10^−3^]).

**Table 2:** Five SNPs showed significant association with one of the tested three cohorts either separately or/and as a pooled samples.

	Locus	Nearest Gene(s)	A1	A2	Stage	OR	L95	U95	P(Q)	I2	Mod	P
1	**rs2771342**											
	14q11.2	*METTL17* (downstream to *RNASE2*)	C	T	AgP-Ger	1.12	0.99	1.26				0.08
					AgP-NL	0.92	0.73	1.16				0.49
					CP-EA-mod	1.15	1.04	1.28				0.00
					CP-EA-sev	1.13	0.98	1.30				0.08
					Pooled	1.12	1.05	1.19	0.37	0.04	FE	7.41E-04
					CP-Ger	1.19	1.05	1.36				0.01
					Pooled (all)	1.13	1.07	1.20	0.42	0.00	FE	2.84E-05
2	**rs9695213**											
	9p24.1	*MRRF* (upstream to *TTLL11*, downstream *PTGS1*)	G	A	AgP-Ger	1.19	1.00	1.42				0.05
					AgP-NL	1.38	0.99	1.93				0.06
					CP-EA-mod	1.27	1.09	1.48				0.00
					CP-EA-sev	1.22	0.99	1.50				0.07
					Pooled	1.24	1.13	1.37	0.86	0.00	FE	7.91E-06
					CP-Ger	1.01	0.84	1.22				0.88
					Pooled (all)	1.19	1.09	1.30	0.34	0.12	FE	5.77E-05
3	**rs1780616**											
	20q11.23	AL391095.1 - *LBP*	T	C	AgP-Ger	1.14	1.01	1.29				0.04
					AgP-NL	1.25	0.98	1.58				0.07
					CP-EA-mod	1.07	0.97	1.19				0.18
					CP-EA-sev	1.17	1.01	1.35				0.04
					Pooled	1.13	1.05	1.20	0.62	0.00	FE	5.44E-04
					CP-Ger	1.01	0.88	1.15				0.92
					Pooled (all)	1.10	1.04	1.17	0.42	0.00	FE	1.67E-03
4	**rs9271850**											
		*HLA-DQH1*										
			A	G	AgP-Ger	1.53	1.34	1.74				2.54E-10
					AgP-NL	1.66	1.32	2.09				1.75E-05
					Meta-AgP	1.56	1.39	1.75	0.54	0.00	FE	2.52E-14
5	**rs17214512**											
		HLA-*DPA1*										
			A	G	AgP-Ger	1.31	1.12	1.54				9.53E-04
					AgP-NL	1.44	1.07	1.93				1.64E-02
					Meta-AgP	1.34	1.16	1.54			FE	5.14E-05

(AgP-Ger; The German AgP samples, AgP-NL; Dutch AgP samples, Mod-CP; European American moderate CP cases, Sev-CP; European American severe CP cases, CP-Ger; German CP samples, A1 = Effect allele; A2 = Non-effect allele; EAF = Effect allele frequency; Cas = Cases; Con = Controls; OR = Odds ratio; CI = Confidence interval; P(Q) = Cochranes Q P-value; I2 = Heterogeneity index; Mod = Model; RE = Random effects; FE = Fixed effects; P = P-value).

## Discussion

This is the first study to investigate the transcriptome of periodontal tissues from susceptible, resistant and “bone-formation” RILs after infection with the periodontal bacteria *P.g* and *F.n* and at a naive status.

The comparison of gene expression with and without bacterial infection in mice of the resistant RILs, the susceptible RILs and the bone forming RILs, showed that the difference in gene expression in response to bacterial infection was strongest in the resistant RILs. Here, we identified eleven significant DEGs. Among the most upregulated genes were *AMTN* and *CALB1*, which are involved in calcium-binding, a relevant process in bone formation. Likewise, the genes *PAPLN* and *FBN2* are glycoproteins of the extracellular matrix, which is involved in regrowth and healing of tissue. Notably, after bacterial or mock-infection we did not identify significant DEGs in the susceptible and bone formation RILs. This may relate to the small sample size, which putatively caused false negative findings. Otherwise, the functional context of the significant DEGs we identified in the resistant RILs may point to the relevance of processes of tissue regeneration to maintain oral health under bacterial challenge. This may indicate that under particular strong bacterial infection as in our experimental setting, the resistance of bone loss was caused by a particular capacity in the regeneration of tissue integrity that was certainly harmed by the invading pathogens. Correspondingly, in our experimental setting disease susceptibility or resistance did not result from special features of the immune response.

The screening for genes that were differentially expressed between the three RILs after bacterial infection identified a very large number of several hundreds to thousands of DEGs. This could relate to the extensive genetic variability between these RILs, which may have induced “transcriptional noise” that precluded the identification of DEGs relating to a different response to bacterial infection. However, the number of DEGs between the mock-infected RILs of the different phenotype groups was considerably lower compared to the number of genes that differed in expression between the RILs after bacterial infection, indicating that the response to the infection caused most of the observed differential expression. The comprehensive set of genes that were differentially regulated after oral bacterial infection can provide a useful reference for human studies, which relate to bacterial induced oral diseases like PD. More specifically, we note that the chromosomal region at *TTLL11* showed the smallest p-value of association in the CP-AgP meta-analysis together with a significant association in the AgP-only analysis. The chromosomal region at *TTLL11* was previously described to be associated with increased quantities of *P.g*.^1^. However, the causative variants and their putative target genes are unknown and we note that the association also locates upstream to the cyclooxygenase *PTGS1*. However, the independent rediscovery of this locus despite the lack of bacterial data together with the suggestive association with AgP and in the meta-analysis of AgP and CP, argues for a true positive association finding. The missing statistical evidence for the suggested risk loci may generally relate to the relatively small sample size of the available PD analysis samples, compared to other complex common diseases. Given the size limitations of the available PD cohorts, true positive associations of genetic variants with moderate effects or with a lower MAF are unlikely to generate p-values, which pass a correction threshold necessary for thousands of independent tests. Thus, the sample size was a major limitation of the human association study. But similarly, this limitation explicitly argues for a complimentary approach to GWAS, in a manner described in the current work.

Likewise, we identified a suggestive association with the gene *LBP* (Lipopolysaccharide binding protein) that was earlier reported to be associated with CP^4^. The soluble acute-phase protein LBP binds to bacterial lipopolysaccharide (LPS) to elicit immune responses. Although the association at this locus was weak in our study, the independent rediscovery argues for a true positive finding.

In the association study that excluded CP patients, we observed a strong association with the HLA-*DOA/-DAP1* locus, which was reported in a GWAS on CP before. However, this association was only significant in our AgP case-control sample and not in the AgP-CP meta-analysis. This can indicate a false negative finding due to different classification of CP in our study and the previous GWAS. However, a true negative finding cannot ruled out at this stage and this association needs clarification. Likewise the strong association with HLA-*DQH1* was only observed in our AgP case-control sample but not in the CP sample.

In summary, this study generated valuable information by giving independent support for the genetic regions at *DAB2IP/TTLL11*, HLA-*DOA* and *LBP* to be involved in the genetic etiology of PD and propose HLA-*DQH1* and *RNASE2* as novel risk factors for AgP and CP. This demonstrates the utility of RILs of the CC as a controlled model system of bacteria induced PD for the discovery of susceptibility genes of human PD.

## Materials and Methods

### CC-RIL selection and assessment

Experiments were performed at the Department of Prosthodontics, Faculty of Dental Medicine, and Hadassah Medical Center, Israel and at the Department of Clinical Microbiology and Immunology, Sackler Faculty of Medicine, Tel Aviv University, Israel. All mouse experimental protocols used in this project approved by the Institutional Animal Care and Use Committee (IACUC) at Tel Aviv University in Israel, with numbers M-08-044, which adherers to the Israeli guidelines, which follows the National Institute of Health (NIH) of United States of America (USA) animal care and use protocols. Mice were monitored daily by the student for their overall health status. Mice, which were observed to suffer (less movement and activity) from the infection, and based on the consultation with the Veterinarian at the small animal unit, were terminated. Furthermore, based on the approved protocol, mice which lost 10% of their bodyweight between two measured points, or 20% overall, of their initial bodyweight, were also terminated. Termination procedure of mice during assessment based animal suffering or at endpoint of the experiment was based of injection of Ketamine and Xylazine with dose of 100 mg/kg (body weight) and 10 mg/kg (bodyweight), respectively for initial euthanasia, and subsequently by CO2 chamber.

Experimental CC-RILs mice at age 8 to 12 wk of the International Livestock (IL) cohort, were provided by the Small Animal Facility, Faculty of Medicine, Tel Aviv University, Israel, and were assessed for their alveolar bone changes in response to a mixed oral infection with a well-known periodontal pathogen. In the current study we used the mixed infection model using the *P.g*. bacteria as a known key bacteria in periodontitis^24,25^ mixed with *F.n*. as another known pathogenic bacteria from the orange complex^26^. As we showed in our previous studies, the mixed bacteria mimic the periodontal infection in human and induce a prominent bone loss in mice^27^. Briefly, each CC line was divided in an infected and a control group. The mice were treated with Sulfamethoxazole (0.8 mg/ml) in drinking water for a continuous period of ten days, followed by an antibiotic-free period of three days, before oral application of mixed culture of *P.g. (strain 381)* and *F.n. (strain PK1594)* (control groups were treated with PBS and 2% Carboxymethycellulose)^27,28^. 42 days after the last infection, the mice were sacrificed and the alveolar bone volume at the second molar of the left hemi-maxilla was evaluated by micro–computed tomography (µCT)^29^. The CC lines showed significant variation in their response to the mixed infection. While susceptible CC lines showed significant decrease in bone volume (P < 0.05) after infection, the resistant CC lines did not show significant change in bone volume after infection. In addition, a group of CC-RILs showed increase in bone volume and was named “bone formation group”^22^. For each of the phenotypes “resistant”, “susceptible” and “bone formation”, two strains were selected for differential gene expression analysis **(**Appendix Figure 8**)**. Because sex effect on changes in the alveolar bone volume was not observed, both sexes were treated equally. Tissue samples from gingiva, alveolar bone, periodontal ligament and teeth were collected as mixed tissues from two resistant CC lines (TAU-IL188, TAU-IL111), two susceptible CC lines (TAU-IL785, TAU-IL551) and two CC lines that showed bone formation after 42 days of oral bacterial infection (TAU-IL2124, TAU-IL2126). Of each strain, total RNA of two biological replicates were sequenced (RNAseq) after bacterial infection and after mock-infection. In the current study, these are designated as cases and controls, respectively. Table 3 summarizes the experimental setting.

**Table 3:** Number and characteristics of RILs used for differential expression analysis.

Cases-Ctrls sample	n of CC_RILs (names)	phenotype	n_cases	n_ctrls	total
#1	2 (TAU-IL188, TAU-IL111)	resistant	4 (2 per line)	4 (2 per line)	8
#2	2 (TAU-IL785, TAU-IL551)	susceptible	4 (2 per line)	4 (2 per line)	8
#3	2 (TAU-IL2124, TAU-IL2126)	bone formation	4 (2 per line)	4 (2 per line)	8
Total	6 lines		12	12	24

Ctrl; control, n; number, RILs; recombinant inbred lines.

### RNA isolation, RNA-sequencing and Differential gene expression analysis in CC-RILs

Total high molecular weight genomic RNA was purified using the RNeasy kit (Qiagen, Hilden, Germany). RNA quality was assessed using a BioAnalyzer (Agilent Technologies, Palo Alto, CA, USA). Samples with the RNA integrity number (RIN) ≥ 7 was used to prepare a library of template molecules.The mRNA was sequenced with a single-end technology (read length of 75) on NextSeq. 500 platform (Illumina, San Diego, USA). Alignment was performed to mouse genome version GRCm38. Quality control assays and differential expression results, were calculated and visualized in R (version 3.3.1)^30^ using R package DEseq. 2^23^. The significance threshold was taken as Padj<0.05. First, we analyzed the differences in gene expression in response to the different environmental exposure (bacterial infection vs. mock-infection) within each phenotype group (susceptible, resistant or bone formation). For each phenotype group, two independent CC-RILs with the same phenotype were analyzed. From each of these CC-RILs, two biological replicates were included. These two replicates were combined with the other two replicates of the second CC-RIL with the same phenotype, to exclude random effects of a single line, resulting in four samples (see Table 3). Second, between the CC-RILs that developed different phenotypes under the same environmental exposure, we analyzed the differences in gene expression. In a case-only design, the gene expression levels of infected CC-RILs of one phenotype group were compared with the gene expression levels of infected CC-RILs of the other two phenotype groups (e.g. susceptible vs. resistant and susceptible vs. bone formation).

### Biological processes associated with the regulated genes

We tested the general differences between each type of response after bacterial infection (resistant, bone formation and susceptible) and considered the basal gene expression differences of the tested CC lines. Biological processes at Padj<0.05 associated with the generated differentially expressed genes and potential regulatory networks between the generated DEGs and the previous periodontitis QTL in CC mice were defined using the IPA software (QIAGEN Inc. https://www.qiagenbioinformatics.com/products/ingenuity-pathway-analysis). Subsequently, we performed functional network analyses of each gene underlying the most significant pathway.

### Selection criteria of candidate DEGs for association mapping in the human case-control samples

1. DEGs derived from the comparison infected vs. non-infected for each of the three phenotypes (N = 13).
2. The ten most up regulated and the ten most down-regulated DEGs of the comparisons of the different phenotypes after bacterial infection (resistant vs. susceptible vs. bone formation group at infection status) (N = 60).
3. DEGs that were previously reported to be associated with PD in GWAS (N = 15).
4. DEGs that located to the novel QTLs *Perio*3 and *Perio4* (N = 6).

### Human gene homology mapping and annotation

Identified candidate genes in mice were mapped to human genes by using the Genehopper database^31^. Genehopper contains 27,443 orthologous gene mappings between mouse and human (Ensembl)^32^. All regions of the human orthologous genes + /− 200 kb were annotated with GWAS associations with P < 10^−5^ from the NHGRI-EBI GWAS Catalog^33,34^. Currently, the GWAS Catalog contains 150 associations (P < 5 ×10^−6^) with a PD phenotype (Appendix table 5). Moreover, the human orthologues were annotated for expression quantitative trait loci (eQTLs) of the public domain by using Qtlizer (www.genehopper.de/qtlizer), an annotation method of the Genehopper web application, which is integrating data from multiple eQTL databases.

### Participating human studies

The meta-analysis samples consisted of case-control GWAS of German and Dutch AgP^9^ and of European American^2^ and German CP^4^ patients. The German AgP sample (AgP-Ger) included 680 cases and 3,973 controls. Cases were recruited across Germany by the biobank Popgen^35^, University-Hospital Schleswig-Holstein, Germany. Controls originated from North- and West-Germany and were recruited from the Competence Network “FoCus - Food Chain Plus”^36^, the Dortmunder Gesundheitsstudie – DOGS^37^ and the Heinz Nixdorf Recall Studies 1–3^38^. The Dutch AgP sample (AgP-NL) consisted of 171 cases and 2,607 controls. The Dutch cases were recruited from the ACTA (Academisch Centrum Tandheelkunde Amsterdam) and the Dutch controls were recruited from Rotterdam and Wageningen by the B-Proof Study^39^. Inclusion criteria for AgP were ≥ 2 affected teeth with ≥ 30% bone loss in patients <36 years of age; disease phenotype was diagnosed by full mouth dental radiographs. Genotype imputation was performed using 1000 Genomes Phase 3 reference panel^40^. A detailed quality control and imputation workflow is described in^9^. The European-American CP (CP-EA) sample included 958 severe (sev) CP cases, 2,293 moderate (mod) CP cases and 1,909 controls from the Atherosclerosis Risk in Communities (ARIC) Study and were described before^41^. In brief, the patients were classified by the Centers for Disease Control/American Academy of Periodontology (CDC/AAP) consensus three-level classification system^42^. The CDC/AAP taxonomy uses clinical attachment loss (CAL) and PD criteria to define three CP categories as healthy-mild, moderate, and severe CP cases, the first being the control. Genotyping was carried out using the Affymetrix Genome-Wide Human SNP Array 6.0 and the subsequent genotype imputation was performed on the HapMap Phase II reference with individuals of Northern and Western European (CEU) ancestry. The German CP (CP-Ger) sample, which consisted of 993 cases and 1,419 controls from a meta-analysis of SHIP and SHIP-TREND cohorts^43–45^ was used for validation of the results of the explorative meta-analysis and also included in the pooled analysis that comprised all samples. In brief, subjects within the first and the third tertile of proportion of proximal sites with attachment loss (AL) ≥ 4 mm were contrasted after stratification by sex and 10-year age groups. Age-specific tertiles were defined to include severely diseased cases within each age stratum. Thus, also young and severely diseased subjects were captured and included in the third tertile. Otherwise, those in the third tertile would have been the older ones and those in the first tertile would have included the younger ones only. Individuals aged> 60 years were excluded. The identification of these subjects is important as we assume that genetic predispositions might manifest especially in younger age, while in older subjects these effects are overlaid with environmental risk factor associated disease progression. This case-control sample was previously described in detail^4^. Cases and controls were genotyped either with the Affymetrix Genome-Wide Human SNP Array 6.0 or the Illumina Human Omni 2.5 array and imputed on the 1000 Genomes Phase 1 reference.

### Filtering

In the post imputation QC processing, variants were excluded, which passed the following cut-off criteria: AgP: Hardy-Weinberg-Equilibrium P-value (P_HWE_) < 10^−4^, imputation quality (INFO) of <0.8^9^; CP (Germany): P_HWE_ ≤ 0.001, imputation quality (r^2^_HAT_) ≤ 0.3^4^; CP (European-US): P_HWE_ < 10^−5^ (ARIC) and P_HWE_ < 10^−6^ (Health ABC), imputation quality <0.8^2^. Moreover, we filtered out variants with a minor allele frequency (MAF) < 0.05 because our study lacked statistical power to analyze rare variants. The variant sets did not completely overlap between the different genotype data sets that was mainly due to the different human genome references that were used to impute the different data sets. Only variants with genotype data available in each study were analyzed in the meta-analysis.

### Meta-analysis and mapping to DEGs

We meta-analysed the genome-wide association scans of AgP-Ger, AgP-NL, CP-EA-sev, CP-EA-mod and CP-Ger based on the additive model using logistic regression. By default, we applied a fixed effects model. However, for variants with a high degree of heterogeneity, i.e. a P-value of Cochran’s Q P(Q) < 0.05 and a heterogeneity index I^2^ > 0.5, we applied a random effects model instead. Subsequently, we extracted all associations in the range of the candidate genes + /− 200 kilobases and loci with an association of P_meta_ < 10^−3^ were selected.

## Supplementary information

Supplementary data.

## Data Availability

Genotype data for aggressive periodontitis samples and the chronic periodontitis sample with German descent are available upon request from the biobanks PopGen (https://www.epidemiologie.uni-kiel.de/biobanking) and SHIP (https://www.fvcm.med.uni-greifswald.de/index.html), respectively. Summary statistics for CP-EU can also be downloaded (see^2^).
