# Supplementary material for: Translation of mouse model to human gives insights into periodontitis etiology
Source: Sci Rep. 2020 Mar 17;10:4892. doi: 10.1038/s41598-020-61819-0 (PMC7078197; doi:10.1038/s41598-020-61819-0)
Supplement: Supplementary file 1 — Supplementary data. [file 41598_2020_61819_MOESM1_ESM.pdf]

Translation of mouse model to human gives insights into periodontitis etiology

Aysar Nashef[1,2#], Munz Matthias[3,#], Ervin Weiss[4], Bruno G. Loos[5], Søren Jepsen[6], Nathalie van der Velde[7,8], André G. Uitterlinden[7], Jürgen Wellmann[9], Klaus Berger[9], Per Hoffmann[10,11], Matthias Laudes[12], Wolfgang Lieb[13], Andre Franke[14], Henrik Dommisch[2], Arne Schäfer[3*], Yael Houri-Haddad[1*] and Fuad A. Iraqi[15*]

# and *Equal contribution

[1]Department of Prosthodontics, Dental school, The Hebrew University, Hadassah Jerusalem, Israel.
[2]Department of Oral and Maxillofacial surgery, Poriya Medical center, Israel.
[3]Department of Periodontology and Synoptic Medicine, Institute for Dental and Craniofacial Sciences, Charité – University Medicine Berlin, Germany.
[3]Institute for Cardiogenetics, University of Lübeck, 23562 Lübeck, Germany.
[4]School of Dental Medicine, Tel-Aviv University, Tel-Aviv, Israel.
[5]Department of Periodontology and Oral Biochemistry, Academic Centre for Dentistry Amsterdam (ACTA), University of Amsterdam and Vrije Universiteit Amsterdam, The Netherlands.
[6]Department of Periodontology, Operative and Preventive Dentistry, University of Bonn, Bonn, Germany.
[7]Department of Internal Medicine, Erasmus Medical Center, Rotterdam, the Netherlands.
[8]Department of Internal Medicine section of Geriatrics, Amsterdam Medical Center, Amsterdam, The Netherlands.
[9]Institute of Epidemiology and Social Medicine, University Münster, Germany.
[10]Institute of Human Genetics, University of Bonn, Germany.
[11]Human Genomics Research Group, Department of Biomedicine, University Hospital of Basel, Switzerland.
[12]Department of Medicine, University of Kiel, Germany.
[13]Institute of Epidemiology, Christian-Albrechts-University, Kiel, Germany.
[14]Institute of Clinical Molecular Biology, Christian-Albrechts-University, Kiel, Germany.
[15]Department of Clinical. Microbiology and Immunology, Faculty of Medicine, Tel-Aviv University, Tel-Aviv, Israel.

Appendix Figure 1:

Multivariate analysis of gene expression data from periodontal biopsies. Principal component analysis (PCA) score plot with the two first principal components (PC1 and PC2) plotted on the x- and y-axis. **SC**; susceptible control, **SI**; susceptible infected, **RC**; resistant control**, RI**; resistant infection, **OC**; bone formation control, **OI**; bone formation infection.

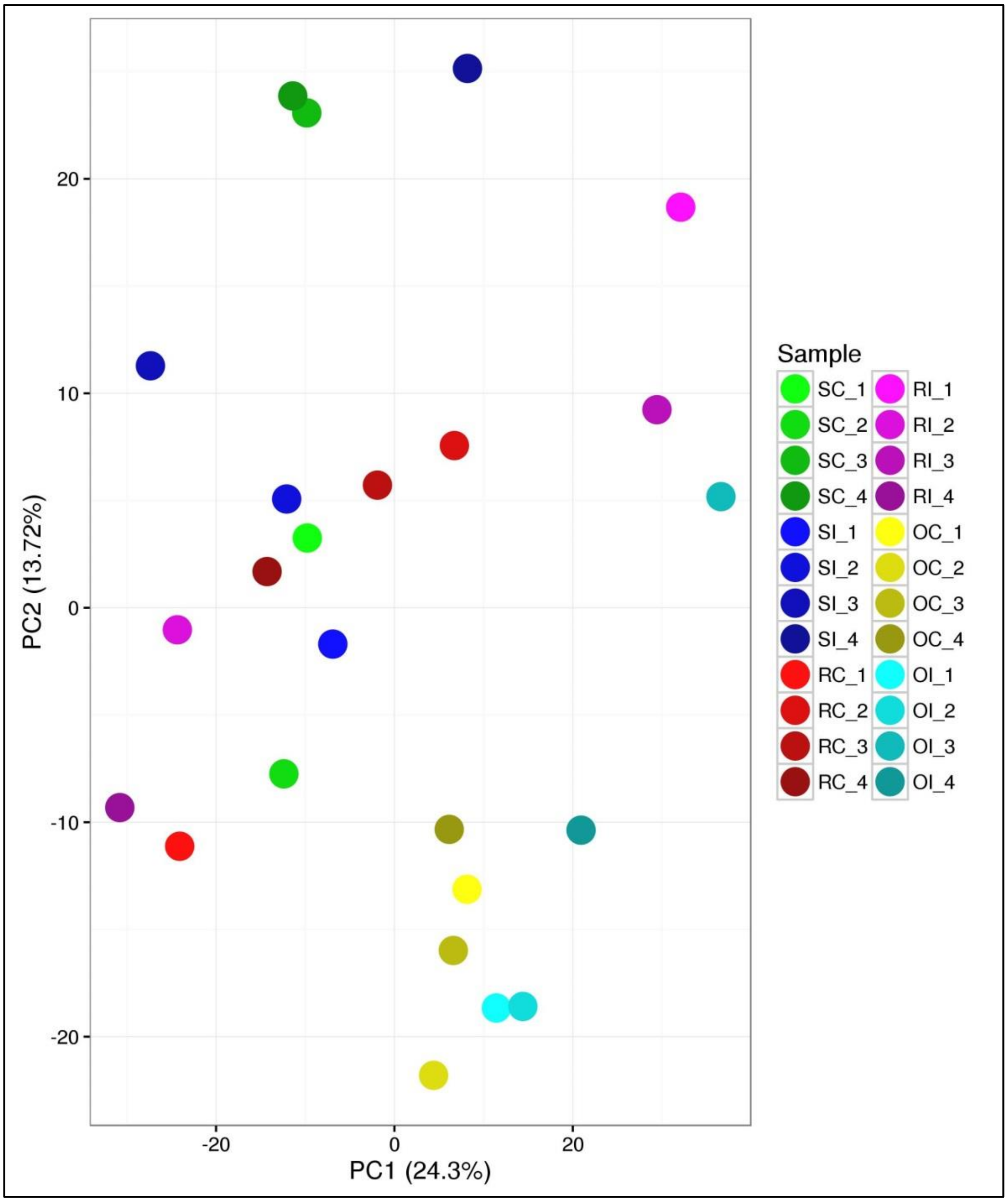

Appendix figure 2: Heatmap of the deferentially expressed genes in the susceptible vs. resistant comparison (A) susceptible vs. bone formation comparison (B) and bone formation vs. resistant comparison(C). **(S; Susceptible, R; Resistant, O; Bone formation, I; Infected, C; Control).**

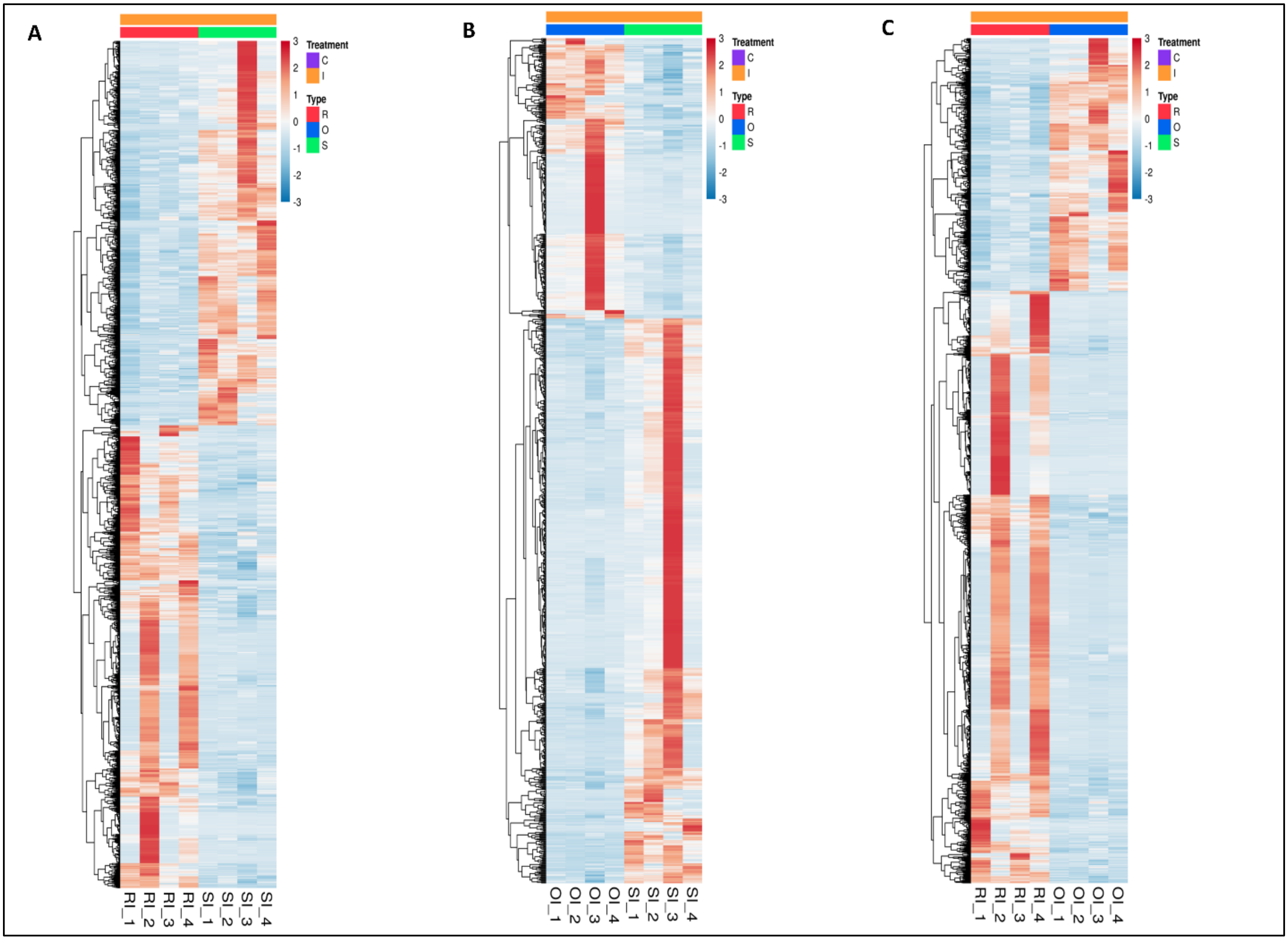

Appendix Figure 3: Pathway enrichment analysis associated with the revealed DEGs between susceptible vs. resistant comparison (A) susceptible vs. bone formation comparison (B) and bone formation vs. resistant comparison(C).

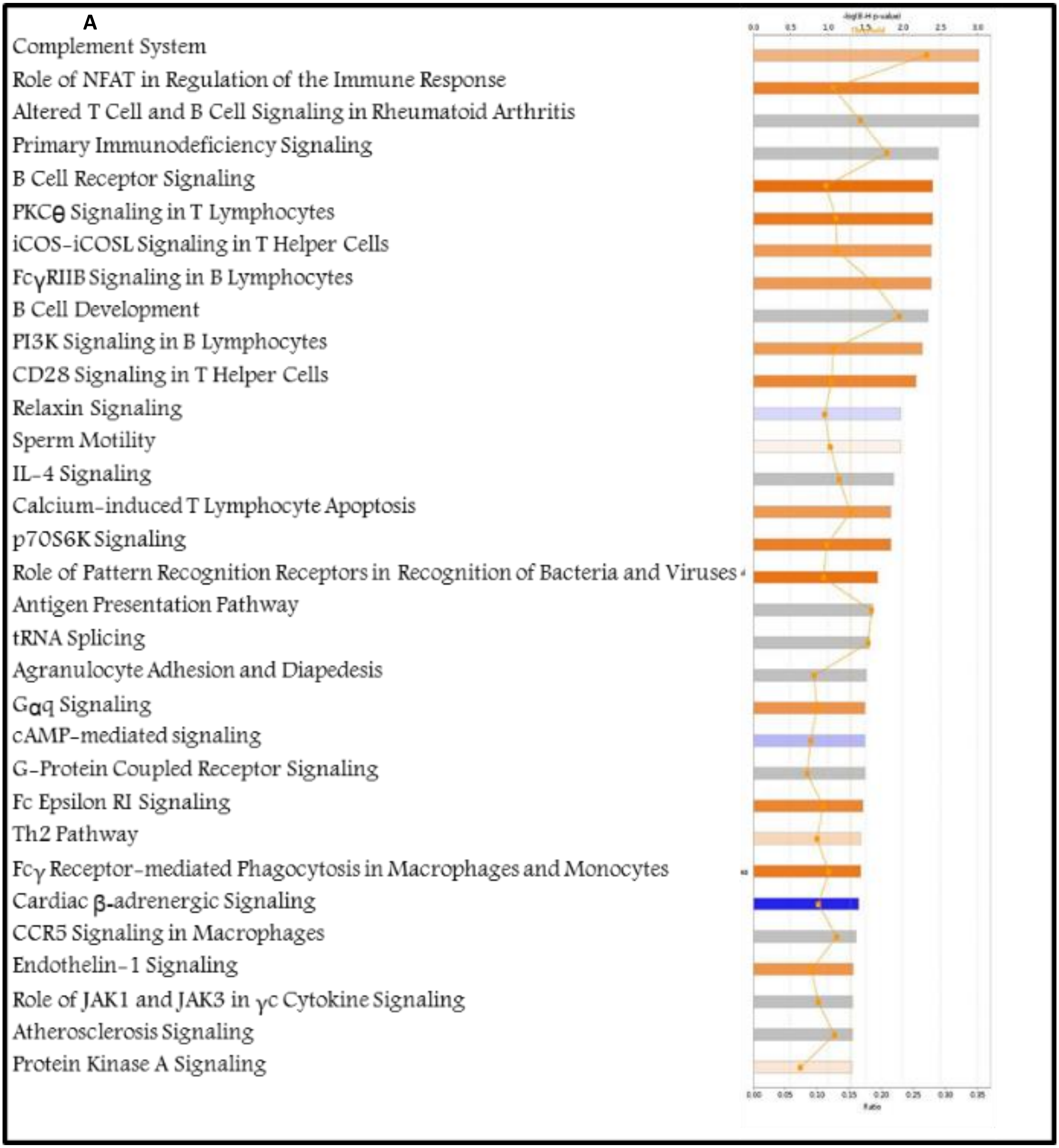

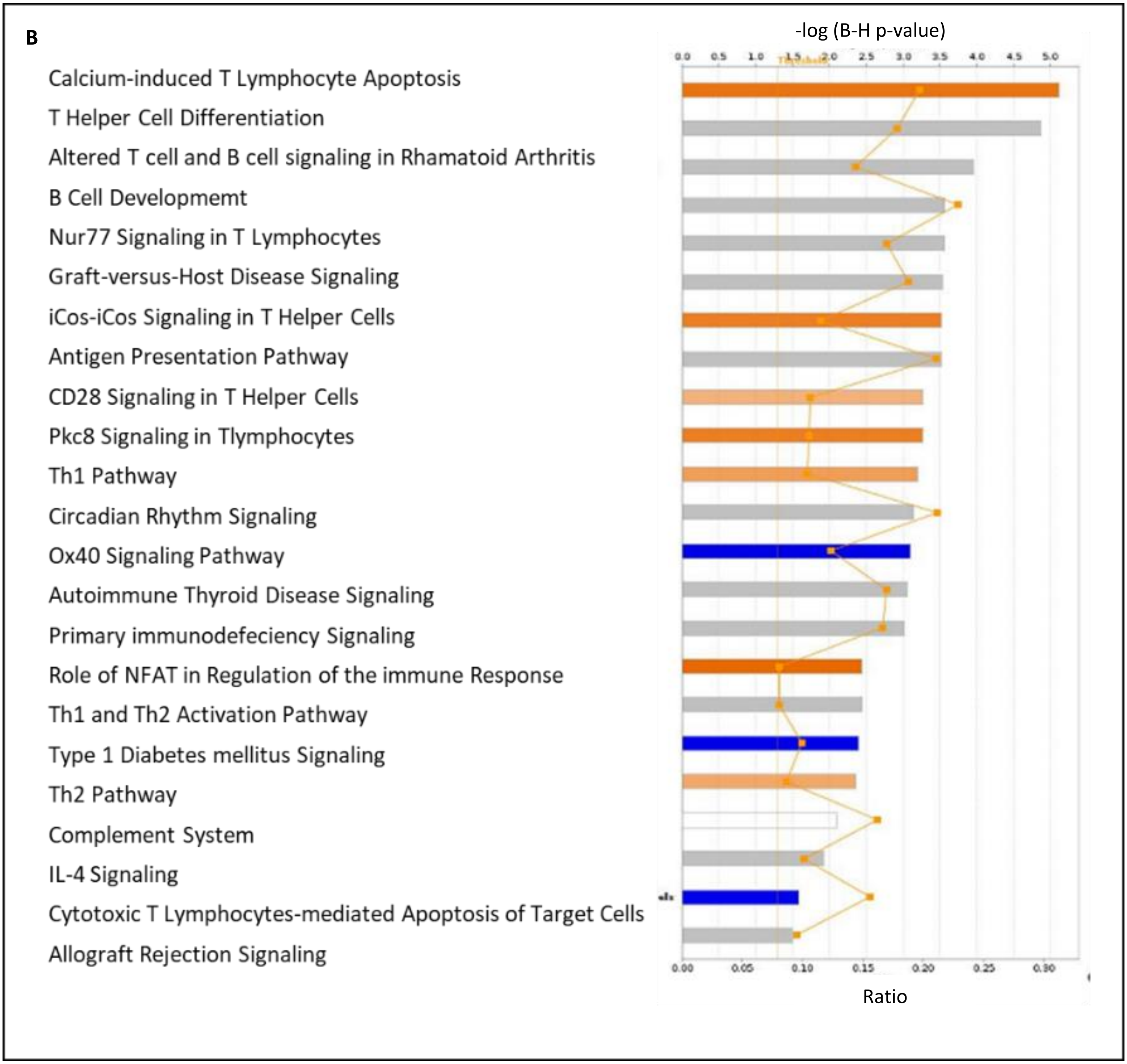
B
-log (B-H p-value)
0.0 0.5 1.0 1.5 2.0 2.5 3.0 3.5 4.0 4.5 5.0
Calcium-induced T Lymphocyte Apoptosis
T Helper Cell Differentiation
Altered T cell and B cell signaling in Rhamatoid Arthritis
B Cell Developmemt
Nur77 Signaling in T Lymphocytes
Graft-versus-Host Disease Signaling
iCos-iCos Signaling in T Helper Cells
Antigen Presentation Pathway
CD28 Signaling in T Helper Cells
Pkc8 Signaling in Tlymphocytes
Th1 Pathway
Circadian Rhythm Signaling
Ox40 Signaling Pathway
Autoimmune Thyroid Disease Signaling
Primary immunodefeciency Signaling
Role of NFAT in Regulation of the immune Response
Th1 and Th2 Activation Pathway
Type 1 Diabetes mellitus Signaling
Th2 Pathway
Complement System
IL-4 Signaling
Cytotoxic T Lymphocytes-mediated Apoptosis of Target Cells
Allograft Rejection Signaling
0.00 0.05 0.10 0.15 0.20 0.25
Ratio

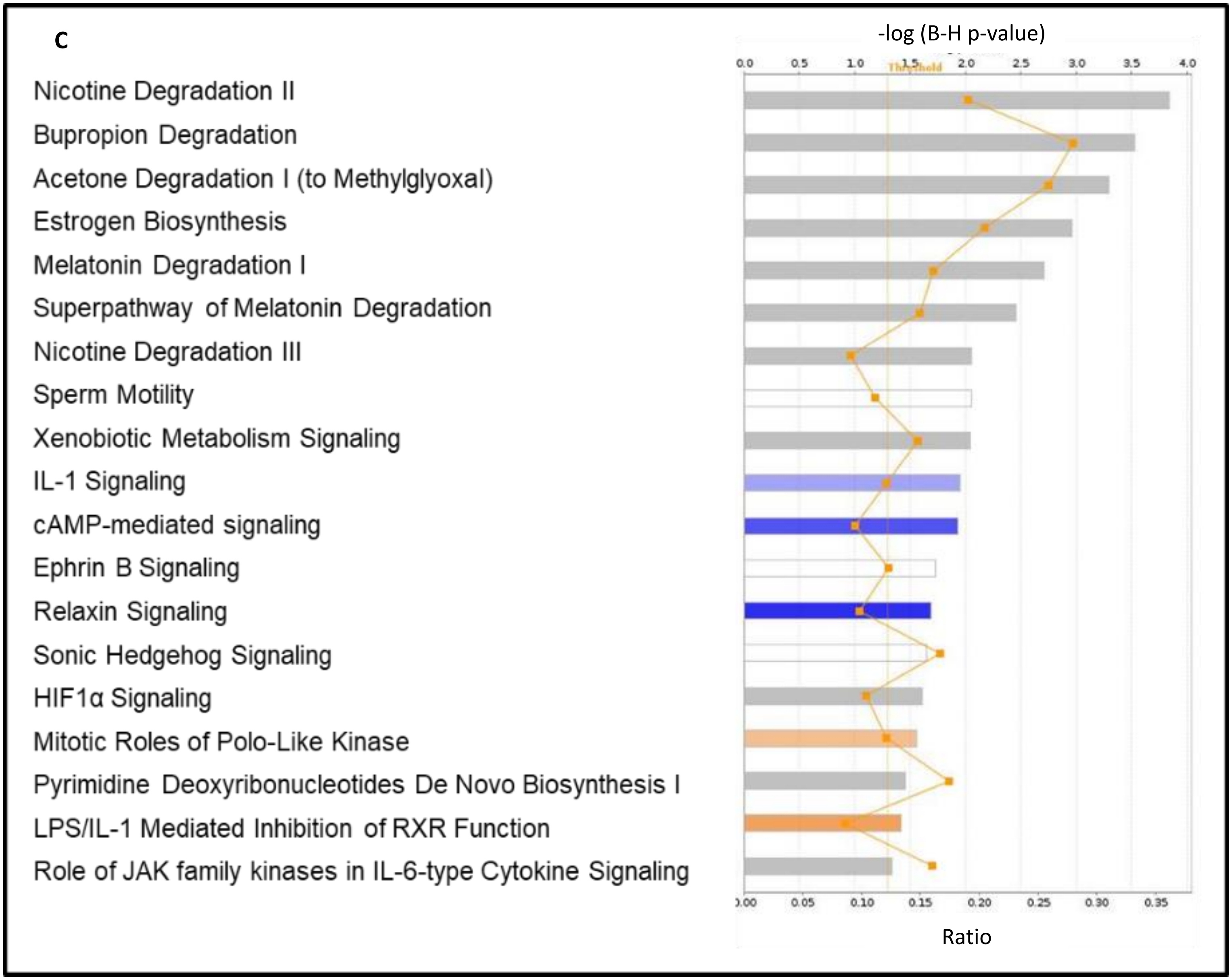
C
-log (B-H p-value)
Threshold
0.0
0.5
1.0
1.5
2.0
2.5
3.0
3.5
4.0
Nicotine Degradation II
Bupropion Degradation
Acetone Degradation I (to Methylglyoxal)
Estrogen Biosynthesis
Melatonin Degradation I
Superpathway of Melatonin Degradation
Nicotine Degradation III
Sperm Motility
Xenobiotic Metabolism Signaling
IL-1 Signaling
cAMP-mediated signaling
Ephrin B Signaling
Relaxin Signaling
Sonic Hedgehog Signaling
HIF1α Signaling
Mitotic Roles of Polo-Like Kinase
Pyrimidine Deoxyribonucleotides De Novo Biosynthesis I
LPS/IL-1 Mediated Inhibition of RXR Function
Role of JAK family kinases in IL-6-type Cytokine Signaling
0.05
0.10
0.15
0.20
0.25
0.30
0.35
Ratio

Appendix Figure 4: A Functional network analysis between enriched pathway at the protein edges level that associated the revealed DEGs between susceptible vs. resistant comparison. Genes are colored based on their related function. **Red;** Complement system**, Yellow;** cAMP mediated signaling, **light red;** CD28 signaling in T helper Cells**, Green;** Fcy receptor mediated phagocytosis in Macrophages**, Blue;** IL4 signaling**, Purple;** Role of NFAT in Regulation of the Immune Response

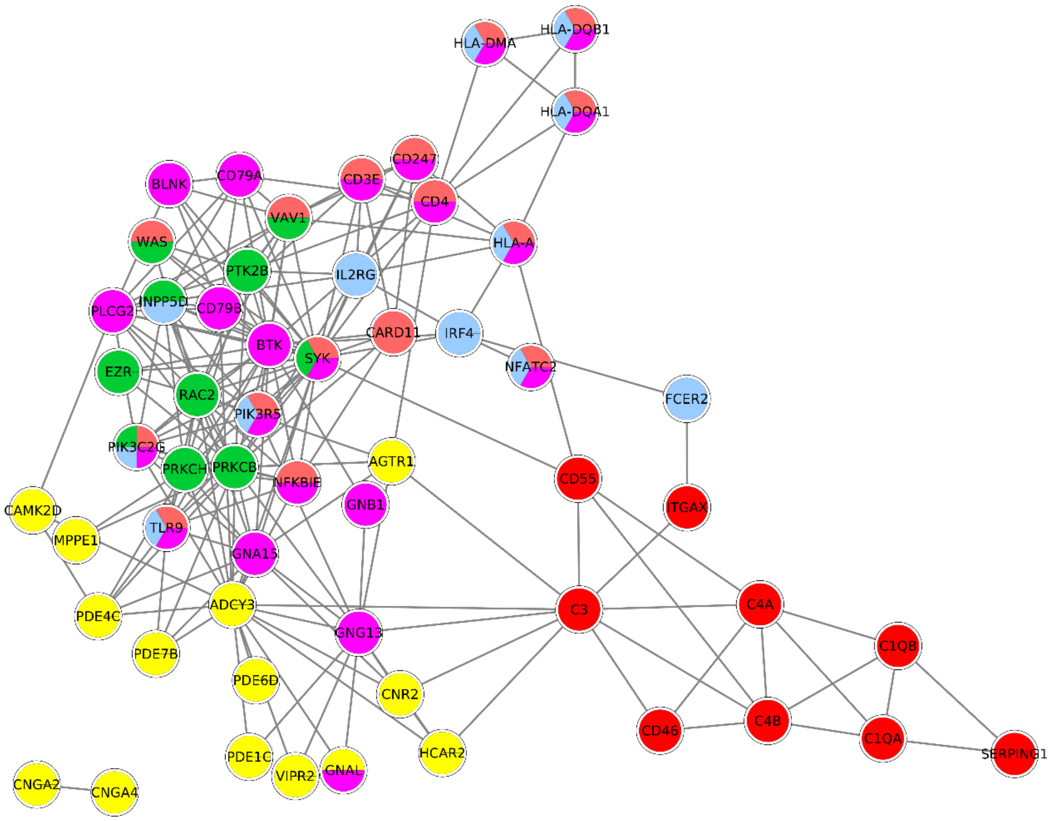

<u>Appendix figure</u> 5: Functional network analysis based protein function level between coding genes underling significant related pathways associated with susceptible vs. bone formation comparison. **Blue**; Antigen Presentation Pathway, **Red** ;Calcium-induced T Lymphocyte Apoptosis, **Green**; CD28 Signaling in T Helper Cells, **Orange**; Complement System, **Purple**; Role of NFAT in Regulation of the Immune Response

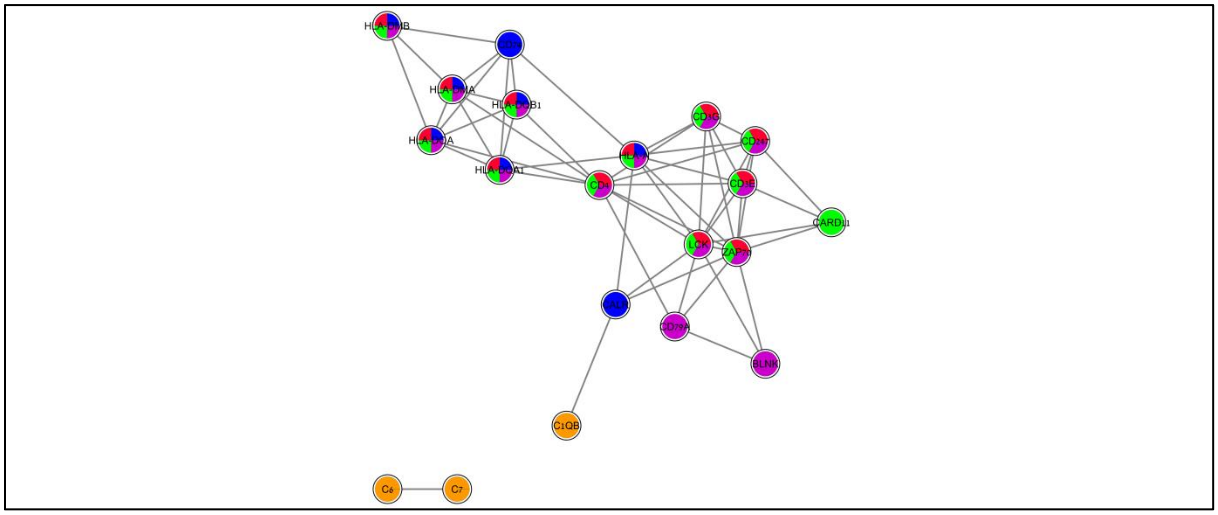

Appendix figure 6:

Functional network analysis based protein function level between coding genes underling significant related pathways associated with resistant vs. bone formation comparison. **Red**: cAMP-mediated signaling, **Blue**: IL-1 Signaling, **Green**: Melatonin Degradation I, **Orange**: Nicotine Degradation II, **Purple**: Relaxin Signaling.

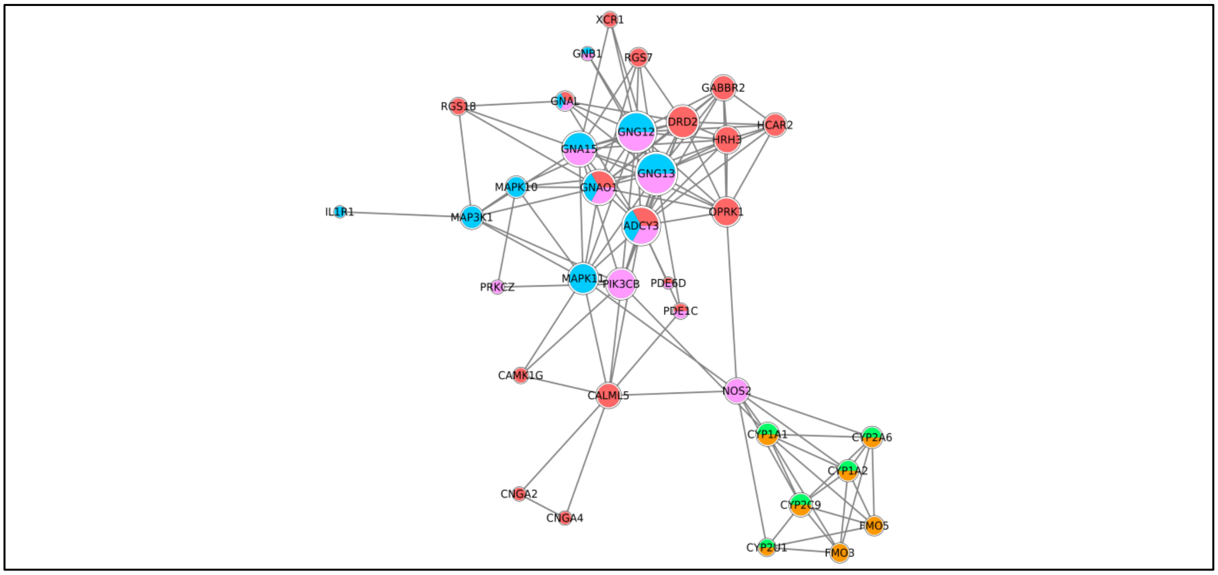

Appendix Figure 7:

Interaction between genes underling previous reported QTL *Perio*3 and *Perio*4 (red nodes) and DEG between susceptible and bone formation generated by IPA tool.

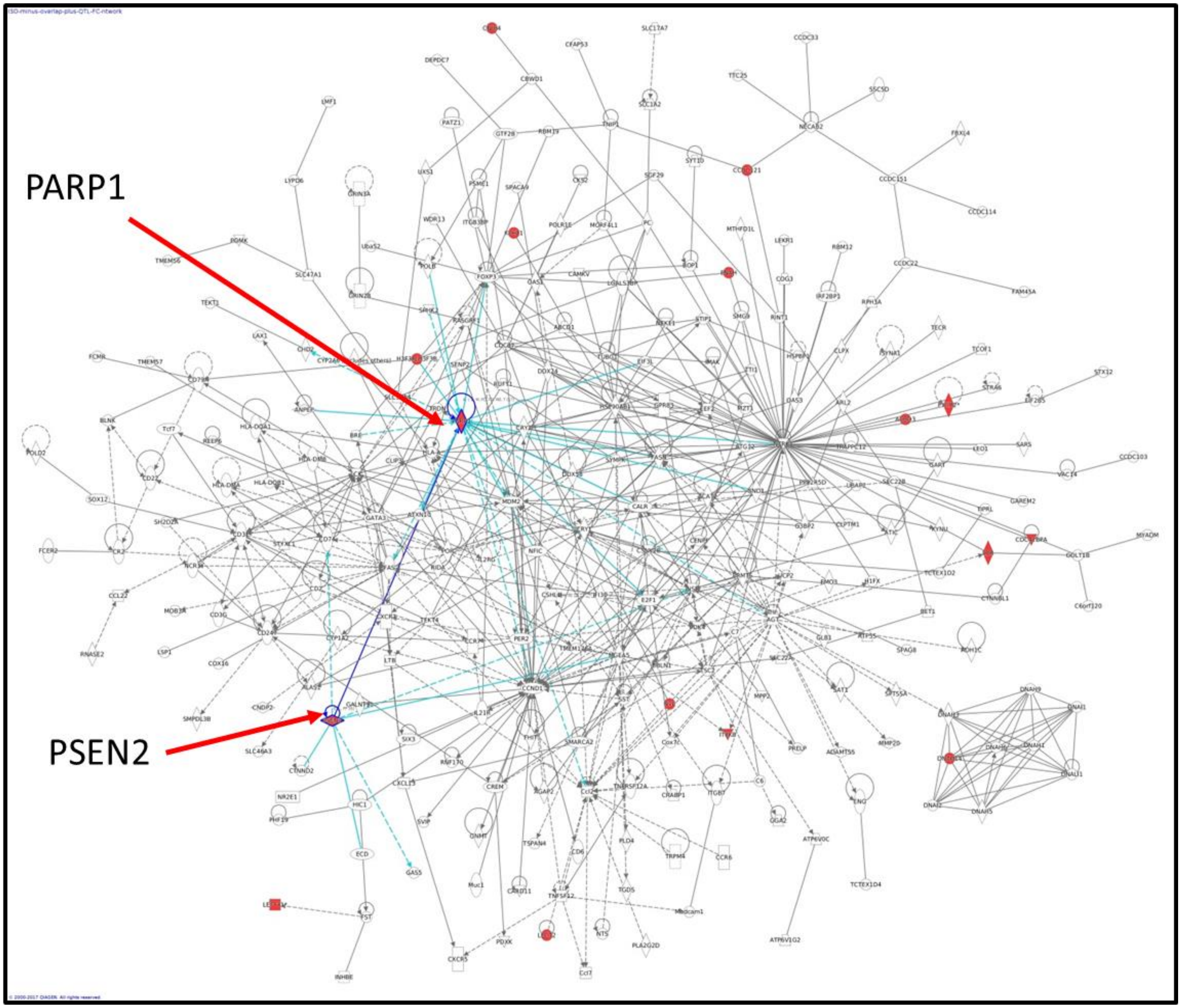

Appendix Figure 8:

Mean of the alveolar bone volume among two resistant CC lines (TAU-IL188, TAU-IL111), two susceptible CC lines (TAU-IL785, TAU-IL551) and two CC lines that showed bone formation after 42 days of oral bacterial infection (TAU-IL2124, TAU-IL2126). Each column represent the mean bone volume of two mice per line per status (infection vs. control).

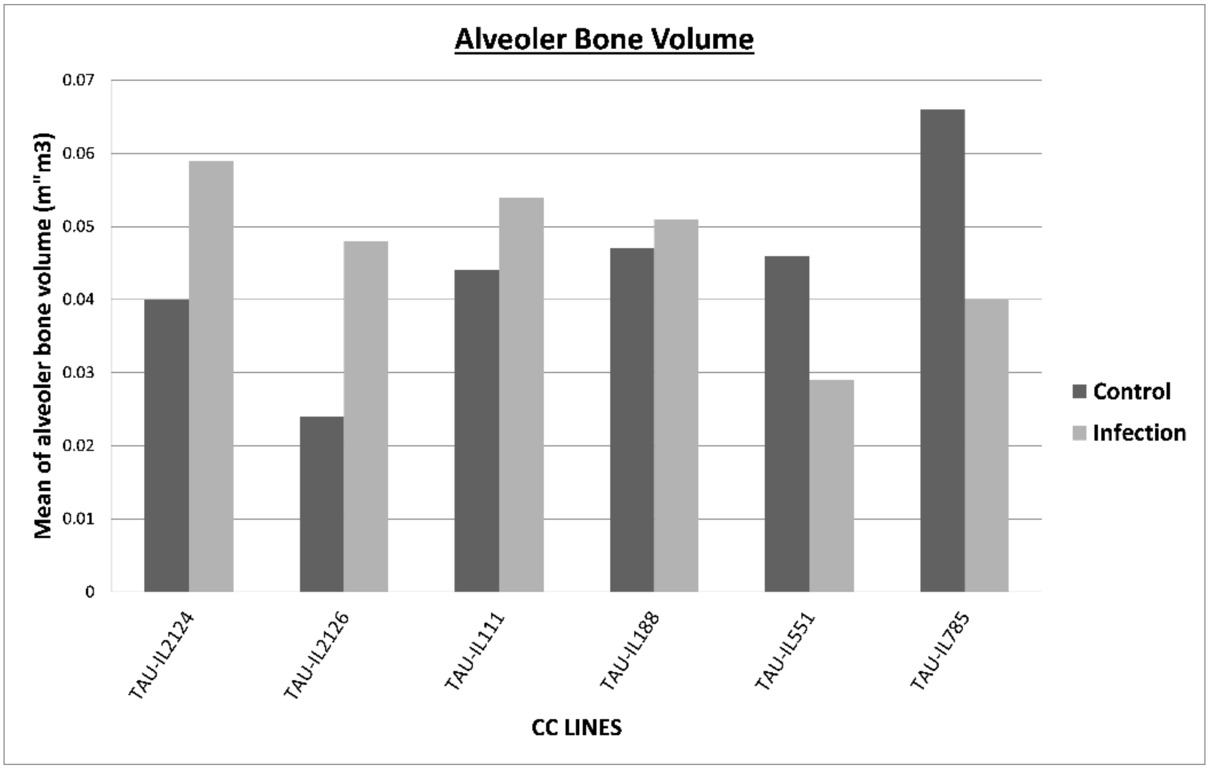

<u>Appendix Table 1:</u> 13 significant DEGs were identified in the phenotype groups “resistant” and “susceptible”. Significant P-values are highlighted in bold letters. All genes except of *Xist* are protein-coding and located on autosomal chromosomes.

		**Resistant**			**Susceptible**			**Bone formation**		
**Mouse Gene**	**Human gene**	**log2FC**	**SE**	**$P_{adj.}$**	**log2FC**	**SE**	**$P_{adj.}$**	**log2FC**	**SE**	**$P_{adj.}$**
Rspo4	*RSPO4*	3,72	0,85	**0.032**	0,41	0,85	0.987	0,05	0,85	1
Mmp20	*MMP20*	7,54	1,45	**0.001**	-0,74	1,51	0.987	4,22	1,45	1
Papln	*PAPLN*	2,84	0,68	**0.050**	0,65	0,69	0.987	0,16	0,69	1
Fbn2	*FBN2*	2,93	0,62	**0.010**	0,03	0,62	0.987	0,13	0,62	1
Calb1	*CALB1*	5,86	1,01	**1,06E-04**	0,20	1,02	0.987	2,23	1,01	1
Amtn	*AMTN*	4,61	1,11	**0.050**	0,49	1,16	0.987	2,82	1,14	1
Gm13762	*OR4C15*	1,63	1,72	NA	-19,62	2,00	**3,82E-18**	-4,98	2,00	**1**
Obp2b	*OBP2A, OBP2B*	-6,52	1,53	**0.048**	0,99	1,53	0.987	0,19	1,53	1
Xist	*XIST (X-chr.)*	-4,97	1,18	**0.050**	0,33	1,17	0.987	0,49	1,20	1
Scd3	*SCD*	-4,76	1,08	**0.032**	1,36	1,07	0.987	-1,72	1,06	1
Elovl3	*ELOVL3*	-5,00	1,19	**0.050**	1,50	1,17	0.987	-1,40	1,17	1
Gm10709	RPL29	0,51	1,17	1	-5,83	1,22	**0.030**	0,87	1,20	1
Bpifa6	*No orthologue*	-7,09	1,32	**5,99E-04**	1,69	1,30	0.987	-2,39	1,31	1

Appendix Table 2:

The table lists the top ten up-regulated and top ten down-regulated genes in each of three comparisons (susceptible vs. resistant vs. bone formation group) with log2FC and Padj of differences. (S; Susceptible, R; Resistant, O; Bone formation).

	**S/R**			**S/O**			**O/R**		
	Gene symbol	padj	Log2FC	Gene symbol	padj	Log2FC	Gene symbol	padj	Log2FC
Up regulated genes	*Obp2b*	7.4E-06	9.0	*Ly6i*	3.8E-05	4.1	*Ces1g*	4.0E-05	2.2
	Sprr1a	9.9E-06	3.1	*Car11*	6.4E-05	2.1	*Klrk1*	1.7E-04	2.8
	Ceacam10	1.6E-05	4.8	*Blnk*	1.6E-04	1.2	*Tnfaip8l3*	2.3E-04	1.5
	Cxcl13	3.4E-05	3.4	*Cr2*	1.7E-04	4.7	*Obp2b*	2.5E-04	7.3
	Ly6i	7.2E-05	3.8	*H2-Aa*	3.4E-04	1.5	*Ms4a4a*	2.9E-04	2.1
	H2-Aa	1.5E-04	1.6	*Smpdl3b*	4.3E-04	2.4	*Nod2*	3.0E-04	2.0
	Cxcl16	1.7E-04	1.5	*Cxcl13*	4.8E-04	3.0	*Mmp3*	3.8E-04	2.1
	Blnk	2.4E-04	1.1	*Prr36*	4.8E-04	1.6	*Cyp2c65*	6.5E-04	4.4
	Gfap	2.5E-04	2.8	*E2f1*	5.9E-04	1.2	*Rnase2a*	6.6E-04	6.8
	Orm1	2.8E-04	1.9	*Foxp3*	6.0E-04	3.9	*Gdpd3*	7.9E-04	3.1
Down regulated genes	*Gm13762*	1.5E-24	-22.1	*Gm13762*	2.1E-07	-14.4	*Kcnc4*	1.8E-10	-4.0
	Plppr1	7.3E-06	-2.9	*Nsun3*	6.9E-05	-1.9	*Gpx6*	1.2E-09	-9.1
	Mmp20	1.6E-05	-8.4	*Cfhr2*	6.0E-04	-7.3	*Bpifb3*	1.8E-07	-8.2
	Calb1	1.7E-05	-5.7	*Nts*	1.0E-03	-5.3	*Cacna1h*	7.6E-07	-4.4
	Msi1	1.8E-05	-2.0	*C7*	1.0E-03	-2.6	*Msi1*	7.6E-07	-2.2
	Acsl6	3.3E-05	-3.3	*Acyp2*	2.0E-03	-3.6	*Vmo1*	1.1E-06	-7.0
	Ric8b	4.9E-05	-2.9	*Ttc32*	3.2E-03	-2.9	*Lhx2*	1.1E-06	-4.6
	Coprs	4.9E-05	-2.3	*Ccl7*	3.3E-03	-3.0	*Calb2*	1.7E-06	-5.8
	Tmem136	4.9E-05	-2.0	*Mettl21c*	3.3E-03	-5.4	*Cdh23*	2.5E-06	-3.3
	Efr3b	7.5E-05	-2.7	*Mettl21e*	3.9E-03	-2.7	*Nrn1l*	2.6E-06	-5.8

Appendix Table 3:

The table lists the genes underlying previous reported QTL (*Perio*3 and *Perio*4) associated with alveolar bone loss induced by P.g and F.n in the Collaborative Cross mouse population.

Associated Gene Name	Chromosome Name	Gene Start (bp)	Gene End (bp)	Human associated gene name	Human Chromosome Name	Human Chromosome start (bp)	Human Chromosome end (bp)
Mir6904	1	180587201	180587267				
Gm17275	1	180802568	180809483				
Cdc42bpa	1	179960472	180165603	CDC42BPA	1	226989865	227318474
Gm38331	1	180145481	180148151				
Adck3	1	180165238	180199602	ADCK3	1	226897536	226987545
Gm38169	1	180021062	180023600				
Gm37336	1	180202303	180212401				
Gm31728	1	180256429	180274963				
Psen2	1	180227004	180263438	PSEN2	1	226870184	226896105
Gm36933	1	180376444	180379514				
Gm37390	1	180372669	180373540				
Itpkb	1	180330485	180424802	ITPKB	1	226631690	226739323
Gm5069	1	180326970	180330550				
Gm37033	1	180357649	180364998				
6330403A02Rik	1	180432387	180483504	STUM	1	226548800	226609214
Gm37984	1	180473355	180477087				
Gm37267	1	180508778	180519727				
Parp1	1	180568924	180601254	PARP1	1	226360691	226408079
Lin9	1	180641150	180690694	LIN9	1	226231149	226309869
Mixl1	1	180693043	180697034	MIXL1	1	226223618	226227054
Gm8101	1	180721614	180722091				
Gm37768	1	180743210	180755836				
Acbd3	1	180726043	180754204	ACBD3	1	226144679	226186730
Gm38293	1	180763497	180768656				
H3f3a	1	180800832	180813943	H3F3A	1	226062707	226072002
H3f3aos	1	180813776	180816859				
Sde2	1	180851127	180868113	SDE2	1	225982702	225999331
Gm20305	1	180854282	180854458				
Gm18036	1	180874878	180875326				
Lefty2	1	180893108	180899103	LEFTY2	1	225936598	225941489
Lefty2	1	180893108	180899103	LEFTY1	1	225886282	225911382
Pycr2	1	180904293	180908088	PYCR2	1	225919877	225924340
Pycr2	1	180904293	180908088	RP4-559A3.7	1	225886696	225924278
Lefty1	1	180935022	180938400	LEFTY2	1	225936598	225941489
Lefty1	1	180935022	180938400	LEFTY1	1	225886282	225911382
2210411M09Rik	1	180952064	180958149				
Tmem63a	1	180942344	180975112	TMEM63A	1	225845536	225882369
Ephx1	1	180976210	181020904	EPHX1	1	225810092	225845563
Gm36961	1	180976158	180976289				
Gm37431	1	181022663	181022773				

Gm37086	1	181026278	181029729				
Gm17967	1	181052528	181053174				
9130409I23Rik	1	181051237	181060667	DEGS1	1	224175756	224193441
Gm37406	1	181062070	181064475				
Gm6606	1	181067596	181076640				
Fgfr3-ps	1	181091129	181092265				
Nvl	1	181087138	181144204	NVL	1	224227334	224330387
Cnih4	1	181144693	181168994	CNIH4	1	224356850	224379459
Wdr26	1	181173228	181211552	WDR26	1	224385143	224437033
A430110L20Rik	1	181225986	181228498				
Rpl35a-ps2	1	181241988	181242320				
Gm8146	1	181282046	181283892				
Gm16539	1	181431723	181432400				
2900069G24Rik	1	181381178	181382488				
Gm16547	1	181400464	181400756				
Cnih3	1	181352628	181460641	CNIH3	1	224434660	224740549
Ccdc121	1	181509633	181511451	CCDC121	2	27625639	27629012
Dnah14	1	181576559	181815774	DNAH14	1	224928422	225399294
Lbr	1	181815335	181843046	LBR	1	225401502	225428925
Gm37664	1	181686430	181690242				
Gm5533	1	181851574	181853189				
Gm38359	1	181967699	181969923				
2700078F05Rik	1	181950132	181953375				
Enah	1	181896384	182019990	ENAH	1	225486835	225653142
Gm37018	1	181986336	181990204				
Gm24836	1	180832769	180832876				
Gm26004	1	181840453	181840568	RNA5SP512	X	111669829	111669948
Gm26004	1	181840453	181840568	RNA5SP61	1	162338643	162338742
Gm26004	1	181840453	181840568	RNA5SP275	8	107884494	107884611
Gm23690	1	181813607	181813700				
Pcdh9	14	93013410	93890679	PCDH9	13	66302834	67230445
Klhl1	14	96105259	96519102	KLHL1	13	69700594	70108493
Gm15515	14	96231953	96232774				
Gm25133	14	96588827	96588956				
4921530L21Rik	14	95881266	95882775				
Gm23509	14	93138030	93138189				
Gm24680	14	94929701	94929826				
Gm24770	14	96945019	96945110				
Gm24043	14	96444955	96445070				
Gm23324	14	94448221	94448351				

Appendix Table 4:

The table lists the 15 genes that were differentially expressed and were previously associated with periodontal disease phenotype with fold change and p values. **(S; Susceptible, R; Resistant, O; Bone formation, OR; odd ratio).**

Gene Symbol	Comparison (log2FC/Padj)	Periodontal disease-related phenotypes /Trait	SNPS (p value /OR or Beta)	PUBMEDID
C1orf87	S/O (3.69, 0.042)	PAL4Q3/Age 20-60 y.	rs17120400 (4.00×10^{-06}/1.69)	24024966
HLA-DOA	S/O (3.04, 0.01)	CDC/AAP/Age 20-60 y.	rs3128935 (9×10^{-6}/2.28)	24024966
BIRC5	O/R (1.5, 0.01)	Mean PAL/Age 20-60 y.	rs17879146 (1×10^{-6}/0.254)	24024966
CCDC13	O/R (-3.22, 0.01)	DPAL/Age 20-81 y.	rs339665 (6×10^{-6}/0.4)	24024966
GLDC	O/R (-1.6, 0.04)	Red complex	rs16924631 (3.00×10^{-06},2.29)	22699663
GPR141	O/R (2.01, 0.047)		rs2392510 (4.00×10^{-06},1.15)	25672891
OTOF	O/R (-3.7, 0.04)	P. g	rs1011108 (2.00×10^{-06},1.79)	22699663
IFI16	S/O (-1.91, 0.03)<br>O/R (2, 0.01)	(PCT1/Socransky Trait)	rs1633266 (3.00×10^{-08},0.93)	26962152
ETNK2	O/R (1.6, 0.04)<br>S/R (1.8, 0.025)	CDC/AAP/Age 20-60 y.	rs2293335 (5×10^{-6}/1.35)	24024966
TTLL11	O/R (-2.1, 1.3×10^{-5})<br>S/R (-1.9, 3×10^{-4})	Microbiota/P. g	rs10760187 (5×10^{-7}/2.07)	22699663
ATP5S	S/O (-1.53, 0.029)<br>S/O (-1.507, 0.03)	PD2	rs3783412 (8×10^{-6}/1.85)	24347629
GRID1	S/R (1.6, 0.015)	Microbiota/A. actino	rs1970525 (4×10^{-6}/2.89)	22699663
LBP	S/R (2.1, 0.022)	DPAL/ Age 20-60 y.	rs11536940 (2×10^{-6}/0.384)	24024966
NIN	S/R (-2.07, 0.005)	PD2	rs3783412 (8.00×10^{-06},1.85)	24347629
VAV1	S/R (1.3,0.04)	(Moderate CPd)	rs3826782(8.00×10^{-07},2.01)	23459936

Appendix Table 5:

The table list the reported GWAS result at ($P<9\times10^{-6}$) based on available GWAS data from GWAS catalogue as of May, 2017. In total, 13 periodontal related phenotypes, 150 mapped genes 198 reported genes are listed.

PUBMEDID	DISEASE/TRAIT	REGION	REPORTED GENE(S)	MAPPED_GENE	SNPS	P-VALUE	PVALUE_MLOG	P-VALUE (TEXT)	OR or BETA
25256105	Aggressive periodontitis (sex interaction)	7p15.3	NPY	LOC107986777	rs198712	4.00E-06	5.397940009		2.36
24024966	Periodontitis (DPAL)	3p25.2	IQSEC1	LOC105376955, LOC105376956	rs2569991	1.00E-06	6	(Age 20-81 years)	0.2
24024966	Periodontitis (DPAL)	5q23.1	SEMA6A	LOC107986375 - LOC105379138	rs12153048	2.00E-06	5.698970004	(Age 20-81 years)	0.3396
24024966	Periodontitis (DPAL)	3q26.31	NLGN1	NLGN1	rs11709498	6.00E-06	5.22184875	(Age 20-81 years)	0.1549
24024966	Periodontitis (DPAL)	3p22.1	CCDC13	CCDC13	rs339665	6.00E-06	5.22184875	(Age 20-81 years)	0.4063
24024966	Periodontitis (DPAL)	15q11.2	SNRPN	NPAP1 - SNRPN	rs8030136	6.00E-06	5.22184875	(Age 20-81 years)	0.1934
24024966	Periodontitis (DPAL)	3p26.1	SETMAR	SUMF1	rs2587949	8.00E-06	5.096910013	(Age 20-81 years)	0.1432
24024966	Periodontitis (DPAL)	20q11.23	LBP	LBP	rs11536940	2.00E-06	5.698970004	(Age 20-60 years)	0.384
24024966	Periodontitis (DPAL)	17q24.3	KCNJ16	KCNJ16	rs12936361	5.00E-06	5.301029996	(Age 20-60 years)	0.2338
24024966	Periodontitis (DPAL)	2q24.3	SCN2A	SCN2A	rs16850317	5.00E-06	5.301029996	(Age 20-60 years)	0.3519
24024966	Periodontitis (DPAL)	18p11.31	TGIF1	BOD1P1, TGIF1	rs12457997	5.00E-06	5.301029996	(Age 20-60 years)	0.1663
24024966	Periodontitis (DPAL)	15q11.2	C15orf2	NPAP1	rs12902137	6.00E-06	5.22184875	(Age 20-60 years)	0.1809
24024966	Periodontitis (DPAL)	8q22.3	LRP12	LRP12 - LOC105375694	rs4734806	6.00E-06	5.22184875	(Age 20-60 years)	0.2075
24024966	Periodontitis (DPAL)	5q23.1	SEMA6A	LOC107986375 - LOC105379138	rs12153048	6.00E-06	5.22184875	(Age 20-60 years)	0.3459
24024966	Periodontitis (DPAL)	13q33.2	DAOA	LOC105370344 - DAOA-AS1	rs17655948	7.00E-06	5.15490196	(Age 20-60 years)	0.1638
24024966	Periodontitis (DPAL)	21q21.3	GRIK1	GRIK1	rs457352	7.00E-06	5.15490196	(Age 20-60 years)	0.2401
24024966	Periodontitis (CDC/AAP)	9p23	C9orf150	LURAP1L - SNORD137	rs1953021	1.00E-06	6	(Age 20-81 years)	1.35
24024966	Periodontitis (CDC/AAP)	17q25.3	TIMP2	TIMP2	rs2009196	2.00E-06	5.698970004	(Age 20-81 years)	1.41
24024966	Periodontitis (CDC/AAP)	1q43	ACTN2	ACTN2	rs12048046	3.00E-06	5.522878745	(Age 20-81 years)	1.52
24024966	Periodontitis (CDC/AAP)	19p13.3	JMJD2B	KDM4B	rs263063	6.00E-06	5.22184875	(Age 20-81 years)	1.63
24024966	Periodontitis (CDC/AAP)	2p14	ETAA1	LOC105374785 - LOC102800447	rs1833219	8.00E-06	5.096910013	(Age 20-81 years)	1.32
24024966	Periodontitis (CDC/AAP)	8p23.1	BLK	LINC00208 - LOC105379242	rs2243407	9.00E-06	5.045757491	(Age 20-81 years)	1.3
24024966	Periodontitis (CDC/AAP)	7q33	FAM180A	LOC105375522 - MTPN	rs13237474	2.00E-07	6.698970004	(Age 20-60 years)	3.05
24024966	Periodontitis (CDC/AAP)	3q25.32	MFSD1	LOC100287290 - MFSD1	rs6802315	3.00E-07	6.522878745	(Age 20-60 years)	1.37
24024966	Periodontitis (CDC/AAP)	4q35.2	FRG1	LOC105377615 - LINC01262	rs13145041	7.00E-07	6.15490196	(Age 20-60 years)	1.65
24024966	Periodontitis (CDC/AAP)	6q25.3	IGF2R	IGF2R	rs78797168	2.00E-06	5.698970004	(Age 20-60 years)	1.75
24024966	Periodontitis (CDC/AAP)	15q25.2	WDR73	WDR73	rs34069323	2.00E-06	5.698970004	(Age 20-60 years)	6.25
24024966	Periodontitis (CDC/AAP)	3q21.1	PARP15	PARP15	rs78411303	3.00E-06	5.522878745	(Age 20-60 years)	1.96
24024966	Periodontitis (CDC/AAP)	8q24.12	SNTB1	LOC105375731 - LOC107986971	rs7819988	3.00E-06	5.522878745	(Age 20-60 years)	1.32
24024966	Periodontitis (CDC/AAP)	8p23.2	CSMD1	CSMD1	rs28455997	3.00E-06	5.522878745	(Age 20-60 years)	1.48
24024966	Periodontitis (CDC/AAP)	1q32.1	ETNK2	ETNK2	rs2293335	5.00E-06	5.301029996	(Age 20-60 years)	1.35
24024966	Periodontitis (CDC/AAP)	5q35.1	ERGIC1	ERGIC1	rs6890783	5.00E-06	5.301029996	(Age 20-60 years)	6.45
24024966	Periodontitis (CDC/AAP)	2q22.1	LRP1B	LRP1B	rs72899866	5.00E-06	5.301029996	(Age 20-60 years)	1.39
24024966	Periodontitis (CDC/AAP)	8p23.1	LINC00208	LINC00208 - LOC105379242	rs2243407	6.00E-06	5.22184875	(Age 20-60 years)	1.33
24024966	Periodontitis (CDC/AAP)	19p13.3	KDM4B	KDM4B	rs11673509	6.00E-06	5.22184875	(Age 20-60 years)	1.66
24024966	Periodontitis (CDC/AAP)	14q21.1	FOXA1	TTC6	rs12587630	7.00E-06	5.15490196	(Age 20-60 years)	2.69
24024966	Periodontitis (CDC/AAP)	2p23.3	GPN1	GPN1	rs111571364	8.00E-06	5.096910013	(Age 20-60 years)	3.46

24024966	Periodontitis (CDC/AAP)	6p21.32	HLA-DOA	HLA-DOA	rs3128935	9.00E-06	5.045757491	(Age 20-60 years)	2.28
24024966	Periodontitis (CDC/AAP)	10p13	ITGA8	ITGA8	rs28643277	9.00E-06	5.045757491	(Age 20-60 years)	1.32
24024966	Periodontitis (CDC/AAP)	12p13.2	PRB2	PRB2 - LOC440084	rs71455379	9.00E-06	5.045757491	(Age 20-60 years)	1.89
24024966	Periodontitis (CDC/AAP)	10q26.3	C10orf91	LOC105378569 - LOC107984282	rs73389468	9.00E-06	5.045757491	(Age 20-60 years)	2.78
24024966	Periodontitis (PAL4Q3)	2q21.1	RAB6C	LOC151121 - LOC105373613	rs7567687	8.00E-07	6.096910013	(Age 20-81 years)	1.32
24024966	Periodontitis (PAL4Q3)	18q12.3	PIK3C3	LINC00907	rs346452	2.00E-06	5.698970004	(Age 20-81 years)	1.32
24024966	Periodontitis (PAL4Q3)	16p13.3	A2BP1	RBFOX1	rs11866781	5.00E-06	5.301029996	(Age 20-81 years)	1.3
24024966	Periodontitis (PAL4Q3)	5q33.1	NMUR2	LOC101927115	rs294958	5.00E-06	5.301029996	(Age 20-81 years)	1.29
24024966	Periodontitis (PAL4Q3)	3p24.3	ZNF385D	HMGB1P5 - LOC100421669	rs17011371	6.00E-06	5.22184875	(Age 20-81 years)	2.09
24024966	Periodontitis (PAL4Q3)	8p23.1	DEFA1	DEFA9P - DEFA10P	rs2738058	6.00E-06	5.22184875	(Age 20-81 years)	1.29
24024966	Periodontitis (PAL4Q3)	18q12.3	RIT2	LINC00907	rs346221	4.00E-06	5.397940009	(Age 20-81 years)	1.31
24024966	Periodontitis (PAL4Q3)	15q23	THSD4	THSD4	rs1442779	7.00E-06	5.15490196	(Age 20-81 years)	1.33
24024966	Periodontitis (PAL4Q3)	8q24.23	FAM135B	FAM135B	rs10875423	9.00E-06	5.045757491	(Age 20-81 years)	1.43
24024966	Periodontitis (PAL4Q3)	5q22.1	CAMK4	RPS3AP21 - CAMK4	rs1370967	8.00E-07	6.096910013	(Age 20-60 years)	2.21
24024966	Periodontitis (PAL4Q3)	5q22.1	WDR36	LOC100129099 - RPS3AP21	rs17132883	2.00E-06	5.698970004	(Age 20-60 years)	2.19
24024966	Periodontitis (PAL4Q3)	15q23	THSD4	THSD4	rs9806183	2.00E-06	5.698970004	(Age 20-60 years)	1.37
24024966	Periodontitis (PAL4Q3)	3p12.3	ROBO2	ROBO2	rs264537	3.00E-06	5.522878745	(Age 20-60 years)	1.35
24024966	Periodontitis (PAL4Q3)	1p32.1	C1orf87	LOC105378760, LOC105378761	rs17120400	4.00E-06	5.397940009	(Age 20-60 years)	1.69
24024966	Periodontitis (PAL4Q3)	8p23.1	DEFA1	DEFA9P - DEFA10P	rs2738058	7.00E-06	5.15490196	(Age 20-60 years)	1.33
24024966	Periodontitis (PAL4Q3)	8q12.3	NKAIN3	NKAIN3	rs2882926	7.00E-06	5.15490196	(Age 20-60 years)	1.56
24024966	Periodontitis (PAL4Q3)	2q21.1	RAB6C	LOC151121 - LOC105373613	rs7567687	8.00E-06	5.096910013	(Age 20-60 years)	1.32
24024966	Periodontitis (PAL4Q3)	22q11.22	VPREB1	IGL	rs11089937	8.00E-06	5.096910013	(Age 20-60 years)	1.33
24024966	Periodontitis (Mean PAL)	9q31.1	ABCA1	LOC102724761, ABCA1	rs4149263	7.00E-06	5.15490196	(Age 20-81 years)	0.056
24024966	Periodontitis (Mean PAL)	11q24.3	ADAMTS15	LOC105369574 - MIR8052	rs12792526	3.00E-06	5.522878745	(Age 20-81 years)	0.2011
24024966	Periodontitis (Mean PAL)	Xq26.1	BCORL1	LOC105373335 - BCORL1	rs2361405	5.00E-06	5.301029996	(Age 20-60 years)	0.0677
24024966	Periodontitis (Mean PAL)	17q25.3	BIRC5	BIRC5	rs17879146	1.00E-06	6	(Age 20-60 years)	0.2549
24024966	Periodontitis (Mean PAL)	12q22	C12orf74	LOC105369905	rs11829373	7.00E-06	5.15490196	(Age 20-60 years)	0.08
24024966	Periodontitis (Mean PAL)	15q25.1	C15orf37	ST20-AS1 - FDPSP9	rs75598935	6.00E-06	5.22184875	(Age 20-60 years)	0.1721
24024966	Periodontitis (Mean PAL)	16q23.3	CDH13	CDH13	rs149740259	9.00E-06	5.045757491	(Age 20-60 years)	0.2412
24024966	Periodontitis (Mean PAL)	5q21.1	CHD1	LOC107986436 - LOC100652833	rs1500251	9.00E-06	5.045757491	(Age 20-81 years)	0.0545
24024966	Periodontitis (Mean PAL)	10q21.1	DKK1	DKK1 - RPL31P44	rs112125027	5.00E-06	5.301029996	(Age 20-60 years)	0.1042
24024966	Periodontitis (Mean PAL)	3p11.1	EPHA3	NDUFA5P5 - ICE2P2	rs12497795	2.00E-06	5.698970004	(Age 20-81 years)	0.0778
24024966	Periodontitis (Mean PAL)	21q22.2	ETS2	LOC400867	rs9979250	4.00E-07	6.397940009	(Age 20-60 years)	0.1497
24024966	Periodontitis (Mean PAL)	7p15.3	FAM126A	FAM126A	rs73082373	6.00E-06	5.22184875	(Age 20-60 years)	0.126
24024966	Periodontitis (Mean PAL)	Xp21.1	FAM47A	LOC105373153	rs113065570	8.00E-06	5.096910013	(Age 20-60 years)	0.0469
24024966	Periodontitis (Mean PAL)	19q13.42	FIZ1	FIZ1 - ZNF524	rs140900046	4.00E-06	5.397940009	(Age 20-60 years)	0.3405
24024966	Periodontitis (Mean PAL)	10q26.13	HMX3	ACADSB - HMX3	rs61862032	9.00E-06	5.045757491	(Age 20-60 years)	0.1957
24024966	Periodontitis (Mean PAL)	Xq26.2	HS6ST2	HS6ST2	rs7892161	9.00E-06	5.045757491	(Age 20-60 years)	0.0413
24024966	Periodontitis (Mean PAL)	Xq11.1	LOC92249	SPIN4 - LINC01278	rs10127000	6.00E-06	5.22184875	(Age 20-60 years)	0.1006
24024966	Periodontitis (Mean PAL)	18q21.2	MBD2	RPL29P32 - MBD2	rs1995138	4.00E-06	5.397940009	(Age 20-60 years)	0.0957
24024966	Periodontitis (Mean PAL)	6q22.31	NKAIN2	NKAIN2	rs77490164	8.00E-07	6.096910013	(Age 20-60 years)	0.1282
24024966	Periodontitis (Mean PAL)	6p22.3	NRSN1	HNRNPA1P58 - NRSN1	rs146696563	4.00E-06	5.397940009	(Age 20-60 years)	0.2396
24024966	Periodontitis (Mean PAL)	3p22.3	PDCD6IP	LOC101928114	rs145744974	4.00E-06	5.397940009	(Age 20-60 years)	0.2942
24024966	Periodontitis (Mean PAL)	19p13.11	PGPEP1	PGPEP1 - GDF15	rs78015699	7.00E-06	5.15490196	(Age 20-60 years)	0.2155
24024966	Periodontitis (Mean PAL)	18q11.2	PSMA8	NPM1P2	rs8097810	4.00E-06	5.397940009	(Age 20-60 years)	0.105
24024966	Periodontitis (Mean PAL)	10q23.31	PTEN	LOC107984250 - LOC105378414	rs149784093	3.00E-06	5.522878745	(Age 20-60 years)	0.2236

24024966	Periodontitis (Mean PAL)	20q12	PTPRT	PTPRT	rs7345986	9.00E-06	5.045757491	(Age 20-60 years)	0.0637
24024966	Periodontitis (Mean PAL)	18p11.32	ROCK1P1	ROCK1P1	rs57440971	4.00E-06	5.397940009	(Age 20-60 years)	0.1413
24024966	Periodontitis (Mean PAL)	15q13.3	RYR3	LOC101928134	rs2676071	7.00E-06	5.15490196	(Age 20-81 years)	0.0443
24024966	Periodontitis (Mean PAL)	17q24.1	SMURF2	SMURF2	rs111854052	9.00E-06	5.045757491	(Age 20-60 years)	0.3228
24024966	Periodontitis (Mean PAL)	18q11.2	SS18	RN7SL97P - SS18	rs9966832	5.00E-06	5.301029996	(Age 20-81 years)	0.1502
24024966	Periodontitis (Mean PAL)	15q25.1	ST20, MTHFS	ST20, ST20-MTHFS	rs36035742	4.00E-06	5.397940009	(Age 20-60 years)	0.1239
24024966	Periodontitis (Mean PAL)	15q23	THSD4	THSD4	rs1442779	7.00E-06	5.15490196	(Age 20-60 years)	0.0543
24024966	Periodontitis (Mean PAL)	19q13.42	ZNF579	ZNF579	rs149546760	5.00E-06	5.301029996	(Age 20-60 years)	0.339
24024966	Periodontitis (Mean PAL)	6q22.31	NKAIN2	NKAIN2	rs13204086	2.00E-06	5.698970004	(Age 20-81 years)	0.1616
24024966	Periodontitis (Mean PAL)	18q11.2	PSMA8	PSMA8	rs11877878	5.00E-06	5.301029996	(Age 20-81 years)	0.0906
24024966	Periodontitis (Mean PAL)	3p14.3	ERC2	ERC2	rs1875110	4.00E-06	5.397940009	(Age 20-60 years)	0.1283
24024966	Periodontitis (Mean PAL)	11q24.3	ADAMTS15	LOC105369574 - MIR8052	rs12792526	4.00E-06	5.397940009	(Age 20-60 years)	0.2189
24024966	Periodontitis (Mean PAL)	14q24.1	ACTN1	ACTN1	rs12050161	4.00E-06	5.397940009	(Age 20-60 years)	0.07
24024966	Periodontitis (CDC/AAP)	8p23.2	CSMD1	CSMD1	rs1540507	9.00E-06	5.045757491	(Age 20-81 years)	1.47
24024966	Periodontitis (CDC/AAP)	15q26.1	RGMA	LOC101927025	rs6497031	5.00E-06	5.301029996	(Age 20-60 years)	1.84
24024966	Periodontitis (CDC/AAP)	8p23.1	BLK	LINC00208 - LOC105379242	rs2243407	6.00E-06	5.22184875	(Age 20-60 years)	1.33
19897590	Periodontitis	9q34.3	GLT6D1	GLT6D1	rs1537415	6.00E-09	8.22184875		1.59
25672891	Periodontitis	6q13	KCNQ5	KCNQ5	rs9446777	5.00E-06	5.301029996		1.22
25672891	Periodontitis	7p14.1	GPR141, NME8	GPR141	rs2392510	4.00E-06	5.397940009		1.15
22699663	Periodontal microbiota	1q42.2	KIAA1804, KCNK1	RNU4-77P - KCNK1	rs11800854	3.00E-07	6.522878745	(Red)	12.3
22699663	Periodontal microbiota	1p22.2	PKN2	LOC105378836 - LOC107985520	rs12032672	1.00E-06	6	(Red)	1.99
22699663	Periodontal microbiota	5q32	FBXO38, HTR4	FBXO38	rs10043775	2.00E-06	5.698970004	(Red)	2.06
22699663	Periodontal microbiota	9p24.1	UHRF2, GLDC, TPD52L3, IL33	UHRF2	rs16924631	3.00E-06	5.522878745	(Red)	2.29
22699663	Periodontal microbiota	4p14	TBC1D1, PTTG2	LOC105374407, TBC1D1	rs10010758	4.00E-06	5.397940009	(Red)	1.91
22699663	Periodontal microbiota	6p21.1	CLIC5, RUNX2	RUNX2 - LOC107986519	rs1932040	1.00E-06	6	(Orange)	2.47
22699663	Periodontal microbiota	8q23.3	CSMD3, TRPS1	CSMD3 - LOC105375710	rs9942773	2.00E-06	5.698970004	(Orange)	2.07
22699663	Periodontal microbiota	1p36.23	CAMTA1, VAMP3	CAMTA1	rs1616122	5.00E-06	5.301029996	(Orange)	1.85
22699663	Periodontal microbiota	14q24.3	FOS, JDP2	LINC01220 - LOC101928377	rs11621969	9.00E-07	6.045757491	(A. actino)	2.46
22699663	Periodontal microbiota	5q34	ODZ2, WWC1	TENM2	rs6885116	1.00E-06	6	(A. actino)	2.57
22699663	Periodontal microbiota	10q23.1	GRID1, MI346, WAPAL	GRID1	rs1970525	4.00E-06	5.397940009	(A. actino)	2.89
22699663	Periodontal microbiota	1q42.2	KIAA1804, KCNK1	RNU4-77P - KCNK1	rs11800854	4.00E-06	5.397940009	(A. actino)	8.12
22699663	Periodontal microbiota	2q31.1	KIAA1715, EVX2, EXTLP2	EXTL2P1 - KIAA1715	rs9287989	4.00E-06	5.397940009	(A. actino)	1.8
22699663	Periodontal microbiota	2p23.3	OTOF, C2Orf70, CIB4	OTOF - C2orf70	rs1011108	2.00E-06	5.698970004	(P. gingi)	1.79
22699663	Periodontal microbiota	9q33.2	TTLL11, DAB2IP	DAB2IP - LOC107987125	rs10760187	5.00E-07	6.301029996	(P. gingi)	2.07
22699663	Periodontal microbiota	10p11.21	FZD8, ANKRD30A	NAMPTP1 - LOC105376496	rs1360573	2.00E-06	5.698970004	(P. gingi)	2.75
24347629	Periodontal disease-related phenotypes	18q12.1	CDH2	CDH2 - ARIH2P1	rs11659841	9.00E-06	5.045757491	(PD1)	2.48
24347629	Periodontal disease-related phenotypes	18q12.2	FHOD3, KIAA1328, TPGS2	FHOD3	rs8094794	6.00E-06	5.22184875	(PD1)	2.17
24347629	Periodontal disease-related phenotypes	8q24.13	HAS2AS, HAS2	LOC105375732 - MRPS36P3	rs3870371	6.00E-06	5.22184875	(PD1)	2.15
24347629	Periodontal disease-related phenotypes	4p15.33	RAB28, BOD1L, NKX3-2, HSP90AB2P	LOC105374494	rs733048	1.00E-06	6	(PD1)	2.4
24347629	Periodontal disease-related phenotypes	11p15.4	NR	GVINP1 - GVINP2	rs12799172	5.00E-06	5.301029996	(PD1)	2.12
24347629	Periodontal disease-related phenotypes	6q22.33	ARHGAP18, LAMA2	LOC102723409	rs7749983	2.00E-06	5.698970004	(PD1)	2.39
24347629	Periodontal disease-related phenotypes	4p15.33	RAB28, BOD1L, NKX3-2, HSP90AB2P	LOC105374494	rs733048	4.00E-06	5.397940009	(PD2)	1.95
24347629	Periodontal disease-related phenotypes	3p23	ZNF860, STT3B, OSBPL10, GPD1L, CMTM8	OSBPL10	rs12630931	6.00E-06	5.22184875	(PD2)	1.89

24347629	Periodontal disease-related phenotypes	14q31.1	SEL1L	LOC107984704	rs12589327	7.00E-06	5.15490196	(PD2)	2.13
24347629	Periodontal disease-related phenotypes	14q21.3	CDKL1, MAP4K5, ATL1, NIN, SOS2, L2HGDH, ATP5S, SAV1	CDKL1	rs3783412	8.00E-06	5.096910013	(PD2)	1.85
25008200	Chronic periodontitis	19q13.32	C5AR1	LOC105372427 - C5AR1	rs7254232	3.00E-06	5.522878745		
25008200	Chronic periodontitis	11q14.1	DLG2	DLG2	rs10501568	9.00E-06	5.045757491		
23459936	Periodontitis	7p15.3	NPY	LOC107986777	rs2521634	4.00E-07	6.397940009	(Severe CPd)	1.49
23459936	Periodontitis	6p21.1	NCR2	LOC100505711 - LOC107986538	rs7762544	8.00E-08	7.096910013	(Moderate CPd)	1.4
23459936	Periodontitis	19p13.3	EMR1, VAV1	ADGRE1	rs3826782	8.00E-07	6.096910013	(Moderate CPd)	2.01
26962152	Periodontal disease-related phenotypes	16p12.3	CLEC19A	LOC105371114, LOC105371113	rs1156327	3.00E-10	9.522878745	(PCT1/Socransky Trait)	1.45
26962152	Periodontal disease-related phenotypes	14q11.2	TRA	TRA	rs3811273	2.00E-09	8.698970004	(PCT1/Socransky Trait)	1.22
26962152	Periodontal disease-related phenotypes	12q14.3	GGTA2P	RAB11AP2 - GGTA2P	rs17184007	7.00E-09	8.15490196	(PCT1/Socransky Trait)	1.35
26962152	Periodontal disease-related phenotypes	13q32.3	TM9SF2	LOC107984558 - LINC01232	rs9557237	1.00E-08	8	(PCT1/Socransky Trait)	1.33
26962152	Periodontal disease-related phenotypes	1q23.1	IFI16	IFI16	rs1633266	3.00E-08	7.522878745	(PCT1/Socransky Trait)	0.93
26962152	Periodontal disease-related phenotypes	3p24.1	RBMS3	LINC00693 - LOC105377009	rs17718700	5.00E-08	7.301029996	(PCT1/Socransky Trait)	1.22
26962152	Periodontal disease-related phenotypes	4p15.32	C1QTNF7	LOC101929095	rs4074082	2.00E-08	7.698970004	(PCT3/Aa Trait)	0.55
26962152	Periodontal disease-related phenotypes	8q24.3	TSNARE	MIR1302-7 - LOC100131146	rs9772881	3.00E-08	7.522878745	(PCT3/Aa Trait)	0.46
26962152	Periodontal disease-related phenotypes	7p11.2	HPVC1	LOC107986739 - HPVC1	rs10232172	2.00E-08	7.698970004	(PCT4)	0.45
26962152	Periodontal disease-related phenotypes	12q24.32	SLC15A4	LOC105370068 - LOC105370069	rs7135417	1.00E-09	9	(PCT5/Pg Trait)	0.44
26962152	Periodontal disease-related phenotypes	12p11.21	PKP2	PKP2	rs6488099	1.00E-08	8	(PCT5/Pg Trait)	0.29
26962152	Periodontal disease-related phenotypes	15q11.2	SNRPN	SNRPN	rs904310	4.00E-08	7.397940009	(PCT5/Pg Trait)	0.39
27601451	Chronic periodontitis (mean interproximal clinical attachment level)	1q42.2	TSNAX, DISC1	TSNAX-DISC1	rs149133391	8.00E-09	8.096910013	(Hispanics)	0.139
27601451	Chronic periodontitis (mean interproximal clinical attachment level)	11p15.1	NELL1	NELL1 - LOC107984320	rs75715012	1.00E-07	7	(Hispanics)	0.045
27601451	Chronic periodontitis (mean interproximal clinical attachment level)	5p15.33	IRX1, LINC01017, LINC01019	LOC105374625 - LOC105374626	rs186066047	2.00E-07	6.698970004	(Hispanics)	0.225
27601451	Chronic periodontitis (mean interproximal clinical attachment level)	6p22.3	LOC645157, RNF144B	TRQ-CTG1-3 - LOC105374958	rs10456847	3.00E-07	6.522878745	(Hispanics)	0.026
27601451	Chronic periodontitis (mean interproximal clinical attachment level)	1q22	ASH1L	ASH1L	rs13373934	5.00E-07	6.301029996		0.173
